# Investigating the Genetic Background of Spastic Syndrome in North American Holstein Cattle Based on Heritability, Genome-Wide Association, and Functional Genomic Analyses

**DOI:** 10.3390/genes14071479

**Published:** 2023-07-20

**Authors:** Anna Neustaeter, Luiz F. Brito, W. J. Brad Hanna, John D. Baird, Flavio S. Schenkel

**Affiliations:** 1 Centre for Genetic Improvement of Livestock (CGIL), Department of Animal Biosciences, University of Guelph, Guelph, ON N1G 2W1, Canada; aneustaeter@lunenfeld.ca (A.N.); britol@purdue.edu (L.F.B.); 2 Department of Biomedical Sciences, Ontario Veterinary College, University of Guelph, Guelph, ON N1G 2W1, Canada; bhanna@uoguelph.ca; 3 Department of Animal Sciences, Purdue University, West Lafayette, IN 47907, USA; 4 Department of Clinical Studies, Ontario Veterinary College, University of Guelph, Guelph, ON N1G 2W1, Canada; jbaird@ovc.uoguelph.ca

**Keywords:** candidate genes, crampiness, dairy cattle, GWAS, periodic spasticity, neuromuscular disorder, QTL detection

## Abstract

Spastic syndrome is a chronic, progressive disorder of adult cattle characterized by episodes of sudden involuntary muscle contractions or spasms of the extensor and abductor muscles of one or both hind limbs. In this study, a case-control genome-wide association study (GWAS) was performed on an adult Holstein cattle cohort. Based on the 50 K and high-density (HD) SNP panel GWAS, we identified 98 and 522 SNPs, respectively. The most significant genomic regions identified are located on BTA9 at approximately 87 megabase pairs (Mb) and BTA7 between 1 and 20 Mb. Functional analyses of significant SNPs identified genes associated with muscle contraction, neuron growth or regulation, and calcium or sodium ion movement. Two candidate genes (*FIG4* and *FYN*) were identified. *FIG4* is ubiquitously expressed in skeletal muscle and *FYN* is involved with processes such as forebrain development, neurogenesis, locomotion, neurogenesis, synapse development, neuron migration, and the positive regulation of neuron projection development. The *CACNA1A* gene, which codes for a calcium channel subunit protein in the calcium signaling pathway, seems the most compelling candidate gene, as many calcium ion channel disorders are non-degenerative, and produce spastic phenotypes. These results suggest that spastic syndrome is of polygenic inheritance, with important genomic areas of interest on BTA7 and BTA9.

## 1. Introduction

Genomic selection has been widely adopted worldwide by the dairy cattle industry and has contributed to great genetic progress in many economically important traits [1]. Intensive selection pressure has been accompanied by high inbreeding levels [1,2,3], creating a situation where the risk of breeding carriers of deleterious traits is higher, due to repeatedly selecting relatively few animals with desirable phenotypes. The identification, isolation, and eradication of genetic disorders in Holstein cattle is pertinent due to strong worldwide pressure for high-producing cows that are minimally affected by welfare issues and cause reduced environmental impact [3].

Spastic syndrome is a chronic, progressive neuromuscular disorder of adult cattle characterized by episodes of sudden involuntary muscle contractions or spasms of the extensor and abductor muscles of one or both hind limbs. These episodes are induced by movement and are particularly noticed when the animal stands up and is adapting to bear weight. No signs of spastic syndrome are exhibited when the animal is lying down. In the later stages, the spasms can involve the epaxial thoracolumbar and neck muscles [4,5,6,7,8,9,10,11,12,13].

Some of the names applied to this syndrome include crampiness, stretches, crampy, barn cramps, periodic spasticity, inherited periodic spasticity, neuromuscular spasticity, bovine biceps femoris neuropathy, and spasmodic extensor hypertonus [5,7,10,11,13,14,15]. The term “standings disease” [10,11,12] has also been used because it appears to be more common in bulls tied by the neck in single stalls. In Europe, the condition has most commonly been called “Krämpfigkeit des Rindes” (i.e., crampiness of cattle) [4,8,16]. Other European designations include “Krampe”, “Stallkrampe”, “Gikt”, “Strek”, “Gliedersucht”, and “Stallkrampf” [16,17]. The diversity of names ascribed to the clinical manifestation of spastic syndrome emphasizes the confusion surrounding its true nature [10].

Spastic syndrome occurs in adult cattle of both sexes [5,6,7,8,9,10,12,13,16,18] and has never been diagnosed clinically in calves [18]. The first signs are usually noticed between 3 and 7 years of age [5,7,8,14,16,18,19] and are most commonly observed in cattle over 6 years of age [5,9]. In a series of 65 cases, 49 (77%) were over 6 years of age, with no cases under 3 years of age [5]. The incidence of spastic syndrome is considerably higher in bulls [5,16,18], and bulls of at least 3 years old are considered to be at greatest risk [9]. The condition is particularly a problem in mature bulls maintained in artificial insemination (AI) breeding units [5,8,11]. In a limited number of AI studs and privately owned bulls, Roberts [8] observed an incidence of 10–30% of mild-to-severe spastic signs in older proven bulls. More recently, the prevalence has been reported to be as high as 30% in older, proven dairy sires [12]. In cows, the condition occurs most commonly in animals between four and eight years of age and the signs are usually milder [5,12]. In dairy cows, the higher producing cows in a herd seem to be the most affected [4,6,16,18]. Spastic syndrome has been reported in most breeds of cattle. However, it primarily occurs in dairy and dual-purpose breeds [5,7,8,9,18,20].

The condition has been reported in the following breeds: Holstein-Friesian [5,6,7,9,10,19,21], Guernsey [5,7,9], Ayrshire [5,6,7,9], Brown Swiss [7,9], Jersey [7,9], Norwegian Red cattle [17,18], Danish Red dairy cattle [18], Simmental [22], Shorthorn [5,7], Angus [7], Hereford [7,12], Bos indicus crossbreds [5], and Holstein crossbreds [19]. Spastic syndrome is a disorder that decreases the welfare and functional lifespan of affected animals. The progression of clinical signs can span from 1 to 10 years, ultimately leading to culling or death [5,6,12]. There is no specific treatment for spastic syndrome. Empirical therapy with muscle relaxants and anti-inflammatory drugs can alleviate pain and discomfort during an acute episode of spasticity [5,6,13,16,20]. Currently, the phenotypic detection of spastic syndrome can be difficult. This is due to the adult onset of the disorder, the mild nature of initial clinical signs, and the possibility of signs being missed during recumbency [12]. Genetic studies on spastic syndrome in cattle are uncommon and there are few estimates of its degree of inheritance in the literature. Therefore, ascertaining preliminary heritability estimates is logical to determine the extent to which spastic syndrome is heritable. Estimating heritability of spastic syndrome as a binary trait (where 0 = unaffected and 1 = affected) assumes an underlying normally distributed disease liability.

Heritability estimates for spastic syndrome were previously reported by Boettcher and Wang [23] for Holstein cattle in Canada, resulting in an estimated heritability of 0.23 in the underlying liability scale and 0.003 in the observed binomial scale, with a population prevalence of 0.11%. No disease criteria were defined by Boettcher and Wang [23], and cattle were relatively young (between the ages of 22 and 34 months) when classified for spastic syndrome, which is not optimal for the detection of a late-onset disorder [5,12]. Most of the evidence suggests that the mode of inheritance of spastic syndrome is autosomal recessive with incomplete penetrance [7,8,14,20], or a polygenic trait [13].

Identifying genomic markers and deriving candidate genes for disorders of unknown inheritance could allow for future genetic testing panels to target known detrimental alleles of specific at-risk populations, such as sires in artificial insemination (AI) stations. Also, creating a candidate gene list in an initial affected population will guide future research to genomic areas of potential interest, and mapping significant SNPs to genes may assist in creating a test panel for spastic syndrome disease risk. A test panel could allow producers to identify which young bulls may be at risk of developing and disseminating spastic syndrome before they are widely used in the population. In this context, a genome-wide association study (GWAS) to screen for areas of interest associated with bovine adult-onset spasticity is an appropriate strategy to uncover genomic regions of interest related to spastic syndrome. Therefore, the main objectives of this study were to: (1) measure the degree of inheritance of spastic syndrome via heritability estimation using all available pedigree information; (2) interrogate both moderate and high-density (HD) single nucleotide polymorphism (SNP) genotypes for the discovery of SNPs associated with spastic syndrome through the GWAS; and (3) perform in silico and functional analyses of significant SNPs obtained from the GWAS of a cohort of affected animals, using HD SNP genotypes to obtain a list of candidate genes for spastic syndrome.

## 2. Materials and Methods

### 2.1. Spastic Syndrome Phenotypic Data

Spastic syndrome disease status was previously determined by two veterinarians (Dr. Hanna and Dr. Baird) via differential diagnosis, where other diseases/disorders presenting with similar clinical signs were evaluated and systematically eliminated. Animals that were at least three years of age and displaying episodes of spasms of the extensor and abductor muscles of the pelvic limb(s) were considered to have spastic syndrome, once other causes for hind-limb spasm had been ruled out, such as evident injury, tetanus, or epilepsy [5]. Additionally, adult cattle displaying signs of other neuromuscular diseases were also excluded to provide a clearer distinction between affected and unaffected animals. Because cattle under three years of age were not included in this study, spastic paresis (or “Elso heel”) was eliminated as a possible confounder since it is a congenital disease usually observed in calves three to six months of age [24,25]. The animals identified as unaffected with spastic syndrome had reached at least eight years of age with no evidence of hind limb spasticity.

### 2.2. Genotypic Data and Quality Control

All animals were genotyped with the Illumina BovineSNP50 BeadChip (Illumina Inc., San Diego, CA, USA). This 50 K SNP panel contained 41,847 SNPs after the quality control following [26], with an average of 51.5 Kb spacing between SNPs. Only autosomal chromosomes and SNPs with known genome positions according to the UMD_3.1 bovine assembly map [27] were used in the present study. The imputed high-density (777 K) SNP panel contained 305,341 SNPs evenly spaced across the genome after imputation from the 50 K SNP panel genotype.

The pedigree data contained 79,613 Holstein cattle from Canada, the USA, Germany, Denmark, Spain, Finland, France, Great Britain, Italy, and the Netherlands. There were 11,227 sires, 51,350 dams, and 9363 founder animals. Within the pedigree, there were 41,264 inbred animals with an average inbreeding coefficient of 0.0441, obtained using the software CFC [28].

### 2.3. Phenotypic and Genotypic Data for The Study Cohort

An initial study population consisted of 24 Canadian Holstein bulls (15 affected, 9 unaffected) and 16 American Holstein bulls (11 affected, 5 unaffected). In total, there were 26 affected and 14 control animals, as summarized in Table 1. 

The phenotypic categories to which animals were assigned were: unaffected, affected mild, affected moderate, affected severe, and uncertain. To add statistical power to the analyses, Lactanet provided phenotypic and genotypic data from additional North American Holsteins diagnosed as “affected” or “unaffected” with spastic syndrome by local veterinarians. This included data for 131 additional affected animals, bringing the total number of affected animals to 157: 19 Canadian cows, 2 American cows, 77 Canadian bulls, and 59 American bulls. Data for 94 more control animals was also provided, bringing the total to 108: 87 Canadian cows, 2 American cows, 12 Canadian bulls, and 7 American bulls. Genotypes for these animals were obtained from Lactanet (www.lactanet.ca; Guelph, ON, Canada). The study animals were genotyped with the Illumina 50 K Beadchip SNP chip (Illumina Inc., San Diego, CA, USA), and imputed to the HD (777 K) SNP panel. The genome-wide accuracy of imputation was over 99%, when using a family- and population-based imputation method (FImpute, [29]).

### 2.4. Heritability Estimation

To estimate the heritability of spastic syndrome, a binomial model was fitted using the ASReml software [30], including the overall mean and the random animal additive genetic effect. The pedigree information of 42,010 Holstein cattle was utilized to calculate the additive relationship matrix for fitting the animal genetic effect. The ASReml software utilizes an approximation likelihood technique, penalized quasi-likelihood (PQL; [30]). The probit link function was used in the generalized linear model [30]. The underlying heritability estimate was transformed to heritability estimates on the observed binary scale using the following transformation [31,32]:

h^o2=h^u2∗w2α∗1−α

where w=e−0.5*z2(2π), ${\hat{h}}_{o}^{2}$ is the heritability estimate on the observed scale, ${\hat{h}}_{u}^{2}$ is the heritability estimate on the underlying scale, *α* is the proportion of affected individuals within the population of interest, *e* is the exponential constant, *z* is the standard normal deviate corresponding to the proportion of affected animals (*α*), and *π* is the mathematical constant pi. 

Using this transformation, heritability estimates for different reported prevalence rates within either a general population of classified Holstein cows of 0.55% [33] or a Holstein sire population within AI facilities of 30% [12] were obtained. In addition, the prevalence of spastic syndrome of 59.25%, i.e., 157 affected animals out of 265 animals, was also used.

### 2.5. Genome-Wide Association Study via Generalized Likelihood Score

Single nucleotide polymorphism (SNP) association analysis was carried out using the Sleuth software (Sargolzaei, University of Guelph, Guelph, ON, Canada), which uses a generalized quasi-likelihood score (GQLS) method to analyze genetic associations between SNPs and the trait considered [34]. The GQLS method can be performed on either a quantitative or a binary trait [34]; spastic syndrome is considered to be a binary trait, where affected animals were scored ‘1’, and unaffected animals were given a score of ‘0’. A logistic regression was used to associate the disease status with the genotypes, treated as covariates and response variables, respectively. Analyses were carried out one SNP at a time, where *X_i_* = (*X*_1_, …, *X_n_*)′, with *X_i_* defined as the phenotypic (disease status) observation of the *i*th animal as 0 or 1, and where *Y_i_* = (*Y*_1_, …, *Y_n_*)′ represents the genotype of the animal, where *Y_i_* = *½* × (the number of alleles 1 in the genotype of animal *i*). With genotypes coded as “0”, “1”, and “2”, the allelic proportions were 0, ½, or 1. The expected SNP allele frequency is represented by *μ*, where *μ* = (*μ*_1_, …, *μ_n_*) = *E*(*Y*|*X*), such that 0 < *μ_i_* < 1. To associate the SNP allele frequency, *μ_i_*, with the phenotype, *X_i_*, the following logistic regression model was defined:

$$\mu =E\left(Y_{i}|X_{i}\right)=\frac{e^{\beta_{0}+\beta_{1}X_{i}}}{1+e^{\beta_{0}+\beta_{1}X_{i}}}$$

where *β*_0_ is a constant and *β*_1_ is the angular coefficient. To verify the association between the marker and the trait, the null hypothesis, *H*_0_: *β*_1_ *=* 0, assumes the marker is not associated with the trait (spastic syndrome), against the alternative hypothesis, *H*_1_: *β*_1_ *≠* 0, where the marker is associated with the trait. 

The 50 K SNP genotypes and the imputed HD genotypes were analyzed with a minor allele frequency (MAF) threshold of less than or equal to 0.00001. This threshold will screen for virtually all possible SNPs of interest, including rare variants. The call rates for individuals and SNPs were both set to 0.90, and a heterozygosity excess greater than or equal to 0.499 was excluded. Significance thresholds were set to 1%, 5%, and 10% chromosome-wise positive false discovery rates (pFDRs). The pFDR was used to increase the sensitivity of correctly detecting SNPs with potentially small effects on the disease, as the pFDR allows for more statistical power than traditional control of family-wise error rates (FWER; [35]). 

To narrow down a potentially large number of significant SNPs identified in the GWAS for the HD data, a post-GWAS MAF threshold of 0.2 was applied to obtain SNPs in relative abundance, as spastic syndrome prevalence is approximately 60%. Additionally, a chi-square test was performed to determine which significant SNPs were significantly different when compared with disease status with a threshold of 0.05. The population proportionate distributions of homozygotic SNPs were calculated in the affected and unaffected population, and the differences of the homozygous states were taken. Then, the absolute difference of the differences in homozygotic status was retrieved to identify the potential polarity of homozygous SNP genotypes and disease status using a threshold of 0.3. This analysis highlights SNPs with one homozygotic variant, which is observed more frequently within affected populations, while the alternative homozygotic variant is more frequent in the unaffected population.

### 2.6. Genome-Wide Association via Random Forest

An alternative GWAS approach based on random forest (RF) regression was used to validate significant SNPs found through the GQLS logistic regression method. Liaw and Wiener [36] developed this non-parametric machine learning method to approach the issue of having a large number of parameters to estimate, i.e., SNP effects, with a small number of observations i.e., spastic syndrome cases and controls. RF regression is a tree-based ensemble machine tool for the classification/regression of multiple variables. Both the trait value and the SNP marker are shuffled to provide robustness against model overfitting [36,37]. The approach is as follows: (a) a random subset of binary observations is selected (i.e., affected or unaffected animals); (b) a random subset of SNP markers is selected; (c) a single tree is created by recursively splitting the subset of SNPs in the subset of samples to form tree nodes; (d) additional animals are sampled with replacements using ‘out-of-bag’ (OOB) data, which will be explained further, and the prediction error of the tree is determined; (e) a forest of trees is generated by repeating steps a–d; and (f) the final SNP variable importance (as VIM%) is obtained by averaging the prediction error values across all trees in the forest [37]. 

To determine the prediction error of the test set, a process called ‘bagging’ is used, where successive trees do not depend on earlier trees and where all trees are independently constructed using a bootstrap sample of the data [37]. Approximately one third of the trees created are left out of the bootstrap sample and used for the OOB error estimate, where a simple majority is taken to obtain the error estimate [36,37]. The weighting of the prediction error was even between disease statuses, and training sets showed lower error rates when regressing on the affected status. Lower errors to correctly describe SNPs implicated with spastic syndrome are adequate as this is a screening study and may include SNPs that are not truly associated with spastic syndrome. To optimize the performance of RF and HD genotype analyses, the big random forest (bigRF) was also implemented [38]. BigRF allows trees in the forest to be grown in parallel on a single machine, or to be built in parallel on multiple machines and then merged into one to decrease computing time and computational load [38]. Once the RF procedure is completed, all SNPs are ranked based on their VIM%. The VIM% is also known as the “mean decrease in accuracy”, where large positive VIM% values indicate a SNP of interest. Essentially, if a SNP with a large VIM% is removed from the forest, resulting in an increase in prediction error, the SNP is considered to be ‘important’ [37]. 

The RF regression was performed using the initial set of animals to estimate the VIM% of SNPs in the HD SNP genotype panel (305,341 SNPs) as the training dataset. The results were used for prediction purposes for the additional 225 phenotyped animals as a testing dataset. The forest was grown to 75 trees, as preliminary research suggests cumulative estimation and prediction error rates do not decrease with an increase in forest size, up to 1500 trees. The forest was replicated 100 times to limit spurious associations. Identified SNPs with occurrence frequencies in the top 50% were classified as genomic areas of interest. All SNPs with a non-zero VIM% were saved and compared with other forest results. Common SNPs from at least two forests were obtained, and average VIM% values were calculated. These ‘significant’ SNPs were then compared with results obtained from the HD SNP genotype panel GQLS regression for possible common areas of significance. The analysis was performed genome-wide to ascertain which SNPs were important on a genomic scale.

### 2.7. Post-GWAS Analyses of Resultant Significant SNPs

Several post-GWAS significance threshold criteria were applied to decrease the number of SNPs for in silico and enrichment analyses. The first post-GWAS criterion applied to the SNPs identified was a MAF equal to or greater than 20% since, as disease prevalence within the study population is greater than 60%, the alleles that contribute to the phenotype observed in spastic syndrome are assumed to be relatively abundant. A chi-square test was performed to determine which SNPs were significantly different from expected frequencies when compared with disease status with a threshold of 0.05. The final threshold placed on the remaining significant SNPs was to obtain the population proportionate distributions of homozygotic SNPs in the affected and control populations. Differences of the homozygotic states were taken per phenotype, and then the absolute difference of these differences was obtained to identify potential polarity of homozygous SNP genotypes and disease status. The homozygous polarity threshold was 0.3.

### 2.8. Mapping SNPs to Genes

The SNP lists generated by the GWAS were mapped using the ‘next-generation sequencing SNP’ tool (NGS-SNP; [39]). The significant SNPs from the GWAS were mapped to genes up to two Mb to the left and to the right of the SNP to ensure thorough gene attainment. The NGS-SNP script uses a list of positions in the bovine genome to return a description of nearby genes. The gene descriptions include gene position, and, if known, model species orthologs and basic gene function information.

### 2.9. Identification of Candidate Genes through Enrichment Analyses

A candidate gene list was created for the HD GWAS. Mapped genes were submitted to different bioinformatic web tools; the primary tool was the Database for Annotation, Visualization, and Integrated Discovery (DAVID; [40,41]). The gene ontology (GO) derived from DAVID was used to obtain an overview of gene function, including biological processes, cellular components, and molecular functions of the genes. Then, using literature information on spastic syndrome, the most relevant biological processes were investigated, including locomotion, muscular and musculoskeletal contraction or movement, neuronal growth/development, cellular/nerve receptors, neuron growth/regulation/transmission, calcium/sodium ion movement, axon and synapse growth/transmission, and synaptic vesicle maturation/fusion/exocytosis [5,8,12], reviewed in Jansen et al. [42]. 

Tissue types such as muscle (skeletal) and nervous (nerve axons and brain) were emphasized in the search [5,6,8,10], reviewed in Jansen et al. [42]. Molecular functions that were incorporated in the search involved ions and ion channel activity. The gene functions were explored further using the National Centre for Biotechnology Information [NCBI; www.ncbi.nlm.nih.gov (accessed on 15 February 2015)] meta-database containing a broad collection of genomic and biomedical databases. The NCBI was utilized for human/mouse ortholog location and verification, gene aliases, and relevant literature regarding genes and gene function. 

Subsequently, Nextbio, a data mining bioinformatics tool, was used to obtain information on the human ortholog of a gene of interest (if applicable), and if it was up- or down-regulated in related human neuromuscular disorders [43]. General information on pertinent human orthologous genes was obtained to identify gene function related to neuromuscular/movement disorders, namely Parkinson’s disease, Huntington’s disease, and amyotrophic lateral sclerosis (ALS). 

### 2.10. Linkage Disequilibrium for Significant SNPs and SNPs within Candidate Genes

Large distances may exist between GWAS-derived significant SNPs and SNPs within the candidate genes, and thus up to two Mb to the left and right of each SNP of interest were considered. Linkage disequilibrium (LD; r^2^ values) were obtained to quantify linkage between SNPs and candidate genes. For a well-established and studied trait, such as mastitis, significant SNPs were mapped to genes of up to 100 kilobase pairs (kb) [44]. However, due to the paucity of genetic knowledge surrounding spastic syndrome, a larger mapping distance was considered to obtain all possible relevant genes. Initially, the exact gene location and length were obtained through the Ensembl database (www.ensembl.org). Haplotype blocks of the candidate genes were created with the HaploView software [45], using the solid spine and r^2^ thresholds to create haplotype blocks within candidate genes. A solid spine was developed to search for a “spine” of strong LD from one marker to another, creating an ‘LD block’, where the first and last markers in the haplotype are in relatively strong LD. HaploView was also used to visualize gene length and intra-gene haplotype blocks.

Subsequently, we obtained syntenic LD of GWAS-derived SNPs and SNPs within candidate genes of interest. As the number of study animals was limited, the animals used to obtain pairwise LD were the 1659 HD genotyped Holsteins, also used as the reference population for the imputation analyses. The r^2^ metric was used to find syntenic linkage with a threshold of 0.2 [46,47].

### 2.11. Mutant Variant List

All SNPs within candidate genes analyses were investigated through NCBI. Variant information, including mutant types and protein consequences of mutants, was derived from the Cattle Genome Analysis Data Repository [48], as well as annotations of the HD SNP panel obtained from the Iowa State University (Ames, IA, USA).

## 3. Results

### 3.1. Heritability Estimates

The underlying heritability estimate for spastic syndrome was 0.41 with a standard error of 0.07. On the observed scale and considering a prevalence of 0.59, the estimated heritability was 0.26. Using a prevalence of 0.30, as in older proven AI bulls reported by Tenszen [12], a heritability estimate of 0.24 was obtained. For a predicted prevalence of 0.6% in the general population of Holstein cows in Canada, the estimated heritability is 0.02.

### 3.2. Genome-Wide Association Study Results

The chromosome-wise pFDR was used. The q-value obtained from the pFDR is the “posterior Bayesian *p*-value”. The Manhattan plots given from Supplementary Figures S1 and S2 (Supplementary Figures S1–S9) truncate significance on the *y*-axis at the –log(q) value of 6 to allow for viewing consistency across all chromosomes. SNPs that exceed the significance threshold of 6 are presented as triangles within Manhattan plots. The distribution of significant SNPs per chromosome is summarized in Table 2. The GWAS resulted in 98 significant SNPs across all chromosomes except BTA10, BTA13, BTA23, BTA24, BTA27, and BTA28, as summarized in Table 2.

Figure S1 presents a genome-wide Manhattan plot for this GWAS; significant SNPs are held within quantitative trait loci (QTL) patterns as well as single-SNP peaks. The following chromosomes had significant SNPs held within QTL patterns, with the approximate location of the QTL pattern indicated in megabase pairs (Mb): BTA7 (20 Mb), BTA8 (65–100 Mb), BTA15 (40 Mb), and BTA22 (40–50 Mb). Table 3 displays the top 10 significant SNPs based on the *p*-value for the 50 K GWAS, highlighting the SNP location and at what pFDR threshold the SNP was found to be significant.

The HD GWAS resulted in 522 significant SNPs across all chromosomes except BTA25 when using the significance thresholds described above. A genome-wide Manhattan plot of this GWAS is shown in Figure S2. Several chromosomes contained peaks with QTL patterns, located in BTA7 (1–20 Mb), BTA8 (60–80 Mb), BTA14 (80 Mb), BTA16 (75–80 Mb), BTA20 holding two peaks (10–20 Mb), and BTA22 (20–40 Mb). To refine the number of significant SNPs, a MAF threshold of 0.2 or greater reduced the number of SNPs to 118 on BTA1, BTA2, BTA3, BTA5, BTA7, BTA8, BTA9, BTA10, BTA12, BTA14, BTA16, BTA17, BTA18, BTA20, BTA21, and BTA22. A chi-square test identified 114 significantly different SNPs, located on BTA1, BTA2, BTA3, BTA5, BTA7, BTA8, BTA9, BTA10, BTA12, BTA14, BTA16, BTA17, BTA18, BTA20, BTA21, and BTA22. In total, 67 significant SNPs met the homozygosity polarity threshold of 0.3 across BTA1, BTA2, BTA3, BTA5, BTA7, BTA8, BTA9, BTA10, BTA12, and BTA14. A summary of SNPs found significant at each threshold can be found in Table 4.

Table 5 highlights the top 10 significant SNPs based on the *p*-value obtained for the HD GWAS and various post-GWAS threshold criteria values. A total of 73 SNPs were in common when comparing the 50 K GWAS with the HD GWAS. The common significant SNP frequency per chromosome is summarized in Supplementary Table S1 (Supplementary Tables S1–S7). After the post-GWAS threshold in the HD GWAS, 10 SNPs remained in common with the 50 K GWAS. Information on the common SNPs after all threshold criteria is given in Table 6.

### 3.3. Genome-Wide Association via Random Forest

The number of SNPs with a non-zero VIM% ranged from 67 to 105 per forest, when 100 forests were grown with 75 trees each, and total OOB estimation and prediction error rates ranged from 25.0% to 47.6%, and 28.4% to 39.1%, respectively. In total, 913 SNPs occurred in at least two forests, up to 19 forests; see Table 7 for a summary of SNPs per chromosome. The following SNPs occurred with relatively high forest frequency: BovineHD0700003818 located on BTA7 at 14 Mb occurring in 19 out of 100 forests, BovineHD0900015016 located on BTA9 at 54 Mb occurring in 9 forests, and BovineHD1600011731 located on BTA16 at 42 Mb occurring in 10 forests.

We identified 16 common SNPs when comparing significant SNPs of the RF regression with the 522 significant SNPs of the GQLS regression, located on BTA7, BTA8, BTA14, and BTA20. These SNPs have a relatively high MAF, ranging from 0.26 to 0.49, and homozygotic polarity scores ranging from 0.20 to 0.47. One common SNP does not have a homozygotic polarity score as there is only one homozygotic state for all tested animals. Table 8 provides summary information on these common SNPs, including post-GWAS threshold criteria values imposed on SNPs found significant with the GQLS regression SNPs. The average maximum VIM% for all significant SNPs was small, ranging from a minimum of 2.69 × 10^−19^% to a maximum of 0.00614%, and common SNPs to the GQLS regression had an average VIM% ranging from 0.00067% to 0.00177%. In general, error rates were lower for SNPs regressed to the affected phenotype, ranging from 0.00% to 23.08% for estimation error rates and 1.53% to 14.50% for prediction error rates. The estimation and prediction error values for SNPs regressed on the unaffected phenotype ranged from 50.00% to 100.00% and 56.38% and 89.36%, respectively. This suggests that RF was able to identify variants implicated with the disease phenotype more accurately.

### 3.4. Post-GWAS Analyses and Candidate Genes

The 67 retained significant SNPs from the GWAS resulted in 1048 genes. Table S2 provides a summary of the distribution of genes per chromosome. Candidate gene lists were created for the HD genotype GWAS, as the HD SNP genotype allows for finer mapping to genes of possible interest. To narrow the number of genes for further enrichment analysis, we screened for key biological functions, as described in the Materials and Methods section. Additionally, genes located within SNP peaks and genes that had significant SNPs within were closely examined for relevant biological function. Two genes, *FIG4* and *FYN*, were classified as important candidate genes.

### 3.5. Candidate Gene List

The 67 retained significant SNPs from the GWAS were located on the following chromosomes: BTA1, BTA2, BTA3, BTA5, BTA7, BTA8, BTA9, BTA10, BTA12, BTA14, BTA18, BTA20, BTA21, and BTA22. In total, the SNPs were mapped to 1048 genes; Table 9 provides a summary of the 7 candidate genes based on statistical significance of associated SNPs and gene function.

Below, the candidate genes are described on an individual basis. The within-gene haplotype blocks (Supplementary Figures S3–S9) are a visualization of the size of the genes and the extent of LD found within. In total, seven SNPs mapped to the voltage-dependent P/Q type calcium channel α 1A subunit gene (*CACNA1A*) on BTA7 at approximately 13 Mb (BovineHD0700003455, which mapped within gene, BovineHD0700004239, ARS-BFGL-NGS-102773, BovineHD0700003962, BovineHD0700004113, and BovineHD0700004235). The *CACNA1A* gene codes for a calcium channel subunit protein in the calcium signaling pathway. This calcium channel is present in the dendrite, neuronal cell body, cytoplasm, and voltage-gated calcium channel complex, and is part of various pathways such as the calcium signaling pathway, the MAPK signaling pathway, and the synaptic vesicle cycle [40,41]. Biological processes of this gene include cation transport, calcium-ion-dependent exocytosis of neurotransmitters, adult locomotory behavior, cerebellar Purkinje cell differentiation, muscle contraction, regulation of neurotransmitter transport and secretion, and glutamatergic synaptic transmission [40,41]. In the peripheral nervous system, this gene is expressed mainly at the neuromuscular junction and mediates the release of presynaptic acetylcholine [49]. There is a 92% shared identity with the human ortholog of this gene, which is down-regulated in Huntington’s disease and up-regulated in Parkinson’s disease and ALS [43,50]. Mutations in this gene have been reported as causal in spinocerebellar ataxia type 6 in humans [51]. This gene has over 1400 SNPs implicated in Parkinson’s disease, tabulated through meta-analyses [52,53].

Two SNPs were mapped to the Ras-related protein Rab-3A gene (*RAB3A*) on BTA7, located at approximately 5 Mb (bovineHD0700001946 and Hapmap59438-rs29012637). The RAB3A protein is involved in calcium exocytosis in neurons, axonogenesis, neurogenesis, regulation of neurotransmitter secretion and transport, and synaptic vesicle maturation and exocytosis [40,41]. RAB3A is associated with synaptic vesicles in their GTP-bound form and dissociates from the vesicle upon depolarization of the nerve terminal [54]. There is a protein–protein interaction with the product of DNAJC5, which may be involved in calcium-dependent neurotransmitter release at nerve endings [40,41]. The bovine gene shares a 99% identity with the human gene and is down-regulated in Huntington’s disease and Parkinson’s disease [43,50]. Kapfhamer et al. [55] reported that *RAB3A* knockout mice have synaptic depression, suggesting that *RAB3A* has a regulatory role in synaptic vesicle trafficking. Additionally, one SNP is implicated in ALS, and 20 SNPs are implicated in Parkinson’s disease through meta-analyses of the *RAB3A* gene [52,53].

There were two SNPs mapped to the microtubule-associated protein 1S gene (*MAP1S*) on BTA7, located at approximately 5 Mb (BovineHD0700001946 and Hapmap59438-rs29012637). *MAP1S* is involved in processes like brain development, neurogenesis, neuron differentiation, neuron projection development, and the execution phase of apoptosis [40,41]. MAP1S is highly expressed in post-natal developing and mature neurons, as well as skeletal muscle (reviewed in Halpain and Dehmelt [56]). There is an 83% identity with the human gene [50]. This gene is down-regulated in Huntington’s disease and Parkinson’s disease [43]. It has been suggested that defects in *MAP1S*-regulated programmed cellular destruction (autophagy) could impact neurodegenerative diseases [57]. Additionally, three SNPs associated with the *MAP1S* gene are also implicated in ALS through meta-analyses [52]. There are 147 SNPs associated with the *MAP1S* gene and Parkinson’s disease through meta-analyses [52,53].

A significant SNP mapped to the *bta-mir-23a-201* gene located on BTA7 at approximately 13 Mb (ARS-BFGL-NGS-102773). This gene does not code for proteins, but rather codes for miRNA. This gene is involved in post-transcriptional regulation of gene expression and possibly acts as a chaperone, and there is translational inhibition or destabilization of target mRNA [40,41]. This gene shares a 99% identity with the human ortholog [50]. One significant SNP was associated with the uncharacterized gene ENSBTAG00000039130 on BTA7, located at approximately 14.5 Mb (BovineHD0700003737). At this time, it is thought to encode a novel protein, or may encode part of the TCP-1 chaperonin family related to heat shock proteins [40,41]. One significant SNP mapped the factor-inducing gene 4 (*FIG4*) gene, located on BTA9 at approximately 41 Mb (BovineHD0900010787). This gene has been described in detail previously. One significant SNP (BovineHD0900010787) mapped to the *FIG4* gene is also mapped to the *FYN* gene, located on BTA9 at approximately 40 Mb.

### 3.6. Associated Candidate Gene Variants

SNP mutant variants within the candidate genes analysis are summarized in Table 10. Several variant types are predicted within candidate genes, and Table 10 also quantifies where variants are predicted to be deleterious.

## 4. Discussion

### 4.1. Disease Diagnostics for Spastic Syndrome

To obtain accurate genomic analyses for spastic syndrome, a universal diagnostic criterion is necessary, as the GWAS results in particular can be skewed if misdiagnosis is present. A first set of adult bulls with phenotypes assessed by experienced veterinarians with respect to spastic syndrome was available. Experienced veterinary diagnostics add validity to the results, as the likelihood for misdiagnosis is relatively low. To increase the statistical power of the GWAS, 225 additional phenotyped animals were added based on observed spasticity by field veterinarians. If the veterinarian is unfamiliar with the clinical signs presented in spastic syndrome, this could lead to misdiagnosis, considering that other disorders can result in limb spasticity.

The broad set of clinical signs used for the diagnosis of spastic syndrome (episodic, involuntary muscle contractions or spasms involving the pelvic limbs that are associated with postural and locomotor disturbances, as well as spasticity) could potentially encompass undiscovered disorders with similar phenotypes, resulting in misdiagnosis [8,14]. Furthermore, misdiagnosis may be present at large because, in the seminal research of spastic syndrome conducted by Becker et al. [7], the diagnosis included “some diagnosis of rheumatism”. Rheumatism can be observed macroscopically through increased synovial fluid retention, although it is known that spastic syndrome shows very little in the form of muscular and joint lesions [7,10,58].

It is important to note parallels between animals and humans when discussing neuromuscular disease diagnostics, especially the susceptibility for misdiagnosis. This is because various human neuromuscular disorders have been more thoroughly investigated in terms of symptomatic progression and possible causality. One study of the retrospective accuracy (the probability of a positive test given that the individual has a condition) of Parkinson’s disease (a human disorder similar to spastic syndrome) was reportedly 82% [59], highlighting the potential risk of misdiagnosis. Diagnostic accuracy is imperative for humans and animals alike, for both treatment and management decisions, but also when screening the genome for areas of interest associated with the disease. Ensuring proper disease definition and training for veterinarians with a universal disease diagnostic criterion for spastic syndrome will allow for greater precision and accuracy in the GWAS and further in silico analyses. In conjunction, reliable diagnostic criteria and industry-supported guidelines for managing the breeding of affected animals would allow facilities that possess affected animals to make effective decisions for the minimization or elimination of spastic syndrome. It has been hypothesized that known affected animals are still being used as sires due to other desirable phenotypes, which could lead to unintentional propagation of the trait if consumers of affected bull semen are not made aware of the condition [6]. The monetary commitment to produce and maintain ideal sires may be a motivating factor in knowingly keeping and continued use of affected bulls. Some possible industry and veterinary supported options could be culling the affected animals, veterinary-supervised pain management plans while the animals are not severely affected, public awareness of affected sires, and/or developing a breeding plan to attempt to decrease future propagation of the disease. Although a larger number of animals with phenotypic records (cases and controls) would be desirable for this study, high-quality phenotypes for spastic syndrome are difficult to obtain because there are other diseases with similar signs that could cause confoundments, as discussed above. In this case-control study, the animals were examined by veterinarians that were experts on spastic syndrome, and animals that were older than the usual onset were only considered diseased if they showed signs. For the controls, only animals above 8 years old without any signs were included to guarantee they were disease-free. Therefore, despite the reduced sample size, these were highly accurate phenotypes. Furthermore, as this was a case-control study, the power of the GWAS was significantly higher than using an observational random sample of animals for the same sample size. However, future studies with larger datasets are recommended to validate the associations identified in this study.

### 4.2. Heritability Estimates

The heritability estimate of 0.41 on the underlying scale and 0.26 on the observed scale indicates that spastic syndrome is moderately heritable within this study population. This is similar to the heritability estimate of 0.23 on the observed scale with a herd prevalence of 30%, reported by Tenszen [12]. Thus, when spastic syndrome prevalence is relatively high, heritability estimates on the observed scale are moderate. However, heritability estimates are higher than what would be expected within the general population of cows.

### 4.3. Genome-Wide Association Studies

The GWAS performed for spastic syndrome assumed no sex linkage, as virtually all reports state an even prevalence between male and female cattle [5,6,7,8,12]. Additionally, both phenotyped populations had a disease prevalence rate of approximately 60%, which is higher than the reported herd prevalence of approximately 30% in older dairy sires [12].

#### 4.3.1. Generalized Quasi-Likelihood Score

The discussion of the GQLS results will focus primarily on the high-density (777 K) genomic results, as SNPs considered significant are more likely to be closely associated with loci near to or within genes that contribute to spastic syndrome. The threshold for significance that was used was a maximum pFDR of 10%. Although the false discovery rate (controlling the proportion of false positives among the set of rejected hypotheses) is adequate in multiple test screening analyses that need higher power, fixing the chromosome-wise rejection threshold to a certain value, as in the pFDR threshold, contributes to a greater power of detection of genomic areas of possible interest [60]. This threshold was used to detect SNPs with possibly a small phenotypic effect, and in turn inflated the number of significantly associated SNPs. There were a number of ‘peaks’ obtained with both screening populations, which will be discussed further, and these preliminary GWAS results indicate that spastic syndrome inheritance is likely not to be monogenic.

Given that the affected animals were accurately diagnosed with spastic syndrome and the disease prevalence was considerable within the test populations, any significant SNPs obtained from the GWAS that truly associate with spastic syndrome would likely not be rare in the analyses. However, initial MAF thresholds for the GQLS method were set low, at 0.00001, to observe clearly all significant SNPs that contribute to a possible QTL, even rare allele contributions. Keeping a low initial MAF allows for all significant SNPs to be highlighted if spastic syndrome inheritance is governed by many genes and genomic regions. However, if causal variants for spastic syndrome are very rare, the limited sample size within this study may prohibit detection of these variants.

The GQLS method for the GWAS was primarily used, as the single SNP regression used is not constrained by the amount of genotypic data, and because the method allows for additive genetic relationships [34]. Placing further threshold criteria on SNPs found significant with the HD GWAS was necessary due to the large number of significant SNPs obtained. As this is one of the first GWAS for spastic syndrome, reducing the number of SNPs for further analysis to those with a higher likelihood of association with the disease would be important to limit unnecessary subsequent analyses with these SNPs. An initial threshold of 0.2 for the MAF was appropriate because the disease prevalence reaches over 60%, so SNPs that associate with genetic areas that contribute to the disease would most likely not be rare. This also eliminates SNPs found to be associative by chance. For the HD GWAS, this threshold decreased the number of significant SNPs by 404 (522 to 118 SNPs), indicating that the vast majority of the initial significant SNPs were relatively rare within this study population. A chi-square test was performed to determine which SNPs found significant through the GWAS with a relatively high MAF were significantly different with respect to genotypic frequencies between the affected and the unaffected populations. In total, the number of significant SNPs decreased by 4 (from 118 to 114 significant SNPs), indicating that virtually all relatively abundant significantly associated SNPs are significantly different between phenotypes. Finally, obtaining SNP homozygotic polarity with respect to the disease status helps to identify SNPs that are possibly associated with genes directly involved in the spastic syndrome phenotype. This analysis decreased the number of significant SNPs from 114 to 67; approximately 58% of the 114 SNPs showed homozygotic distributions of interest.

#### 4.3.2. Comparison of Genotype Densities

Significant SNPs are located on various chromosomes for both genotypic densities. The increase in genetic density allows for the GWAS to be conducted on a finer scale, revealing significant SNPs closer to a possibly causative variant, which in this case appears to be located at approximately 87 Mb. This common peak also adds power to the evidence that this genomic area may be an important contributor to spastic syndrome. When comparing the two different genotypic densities (50 K vs. HD), there were 73 common significant SNPs with an initial threshold of maximum pFDR chromosome-wise at 10%. This indicates that approximately 75% of the significant SNPs found in the 50 K analysis remained significant after imputation (73 out of 98). After narrowing the number of HD significant SNPs using the various post-GWAS threshold criteria described previously, 10 SNPs remain in common when comparing the 50 K and HD GWAS, all of which are within observable SNP peaks within the HD GWAS. This suggests that, after all the post-GWAS threshold criteria imposed on the significant SNPs for the HD analysis, there is still sufficient power of the 50 K panel to detect associative SNPs. In conjunction, comparing the GQLS logistic regression with the RF regression of the HD SNP genotype panel resulted in common genomic areas on BTA7, BTA8, BTA14, and BTA20. These areas reflect significant SNPs that remained with the 50 K analysis, as well as HD analysis after the post-GWAS threshold criteria were imposed (Table 6 and Table 8).

Again, the small initial MAF threshold for all GWAS allows for relatively rare alleles to be identified. The post-GWAS thresholds placed on the HD SNPs decreased the number of significant SNPs per chromosome, and in turn this may decrease possible spurious associations with the spastic syndrome phenotype. In contrast, it may also eliminate rare SNPs that are valid associations in the GWAS. This is one reason why the GWAS findings should be validated with a larger sample size, to possibly discern which SNPs are truly associative with the phenotype observed in spastic syndrome.

If spastic syndrome is single allelic and the causative allele is in the SNP panel, or in complete or very high linkage with the causative variant in either SNP panels, common significant SNPs should arise. The reduced overlap observed across both genomic densities may be a function of a low power-of-detection due to the small sample size or it could be that more than one area in the genome is responsible for the phenotype observed with spastic syndrome.

#### 4.3.3. Random Forest

In total, 75 trees per forest were grown, as similar error rates were obtained for forests of up to 1500 trees. Decreased computing time and computational load for the RF regression were obtained through minimizing the number of trees per forest while maintaining minimal error rates. Additionally, 100 replicates of the RF regression were obtained to avoid spurious associations due to sampling within the forest, as the RF regression can be prone to upward bias due to variable selection being random [37,61]. The RF regression obtained SNPs with a non-zero VIM% for all forests. As this is a screening study, it was necessary to analyze all SNPs that appeared to be significant, but not spuriously so; therefore, the SNPs with a non-zero VIM% that occurred in at least two forests were retained, as their presence in at least two forests may decrease the chance that these significant SNPs are anomalous.

As the average VIM% for all SNPs obtained with a non-zero VIM% was small, the frequency of forest occurrence was an alternative measure of the importance of the SNP. SNPs that occurred within approximately the top 50% regarding forest frequency were highlighted. The SNP that occurs most frequently, BovineHD0700003818, is located on BTA7 at approximately 14 Mb, and is within the bounds of the QTL-like peak at 1–20 Mb obtained from the GQLS regression method. This is validation of the QTL-like peak within BTA7. Additionally, the SNP BovineHD0900015016 occurs in nine forests and is located on BTA9 at approximately 54 Mb. Although the QTL-like pattern located at approximately 87 Mb is relatively far from this significant SNP, there are significant SNPs within the chromosome for all GQLS analyses at approximately 40–50 Mb.

The SNPs with a non-zero VIM% from at least two forests were then compared with the 522 significant SNPs obtained from the GQLS regression with the HD SNP genotype panel. In total, there were 16 common SNPs, and those that were common tended to have higher MAF and homozygous polarity scores (Table 8). The RF regression appears to be relatively effective when combined with the GQLS regression to screen for associative SNPs that are not rare.

The generally large error rates for both the prediction and estimation of the RF regression suggest that this method for regression is not sufficient for association analyses, but appears to be sufficient to validate the GQLS regression results with this study population (Table 8). The weighting of the prediction error was even between disease statuses, and the lower error rates for variants regressed to the disease phenotype suggest that RF can more accurately identify SNPs that are associated with spastic syndrome. The first set of animals was used to estimate the SNP VIM%, as diagnostics may be more accurate within this cohort, and an increase in phenotyped animals for RF estimation may decrease error rates for both estimation and prediction values. One common SNP between both regression methods (BovineHD0700006040, located at approximately 21.9 Mb) appears close to a QTL-like pattern on BTA7, located at approximately 1–20 Mb (Figure S2). This SNP has a relatively high MAF (0.381), whereas the homozygotic polarity score of this SNP is relatively low at 0.18. The results from the RF regression may be validating an area of significance also obtained with the GQLS regression within BTA7.

### 4.4. Mapping SNPs to Genes

Due to the significant lack of understanding of spastic syndrome, a mapping distance of up to two Mb to the left and right of the SNP of interest was used. If genes are mapped to significant SNPs at a relatively large distance, as long as ‘useful’ linkage (i.e., r^2^ ≥ 0.2) exists, genes of interest will remain relevant. Direct syntenic linkage was obtained between significant SNPs and SNPs within genes of interest (Tables S3–S7), including haplotype pairs that are farther than two Mb apart. This is because some variants within candidate genes exist farther than the mapping threshold of two Mb, but as they were within the candidate gene the direct linkage was still obtained. Additionally, an understanding of the biological nature of spastic syndrome is necessary to screen this list for appropriate genes for further consideration. The 26 common genes obtained for BTA9 at approximately 40 Mb indicates that this area is likely implicated with spastic syndrome. The two common candidate genes, *FYN* and *FIG4*, have functions of great interest with regard to spasticity, and will be discussed further. Even at relatively large distances there was moderate LD between haplotype pairs, which adds validity to the following candidate genes, as again some genes exist relatively distant from the significant SNP from the GWAS.

Table 10 quantifies the number of predicted deleterious mutations within each candidate gene. The variants detected within candidate genes that are predicted as deleterious may guide future research into the genetic and pathophysiologic nature of spastic syndrome.

The population prevalence of spastic syndrome was approximately 60%, and the GWAS results indicate that there are significant SNPs with a moderate-to-high MAF, along with SNPs that have polarized homozygotic distributions when looking at disease status (Table 9). Spastic syndrome is likely not monogenic, due to numerous genomic peaks obtained from the GWAS. The 67 retained significant SNPs map to seven potential candidate genes on BTA7 and BTA9. Several candidate genes listed below are implicated in degenerative neuromuscular disorders in humans and are likely not causal for spastic syndrome. However, these candidate genes may still contribute to the disorder through unknown cellular mechanisms, especially if the inheritance of spastic syndrome is multi-genic. Two genes exist in both candidate gene lists (*FIG4* and *FYN*) and have been discussed previously.

Mitochondrial Function. It is proposed that a MAP1S deficiency contributes to a reduced ability to recognize defective mitochondria and commence cellular destruction [57]. A mouse knockout study of the *MAP1S* gene reported increases in mitochondrial size and frequent rupture of the outer membrane, followed by a progressive, lethal aggregation of mitochondria [57]. These knockout mice exhibited no obvious abnormalities in development or reproduction. The spastic phenotypes of the knockout mice were progressive in severity, much like what is observed in spastic syndrome. There are no cellular abnormalities reported with spastic syndrome, thus *MAP1S* is likely not causal for this disorder [10]. However, protein–protein interactions could occur with a mutant *MAP1S* that could contribute to the progressive, non-degenerative phenotype observed in spastic syndrome through currently unknown mechanisms.

Calcium Channel Function. Calcium channels play an important role in the regulation of a variety of cellular functions such as membrane depolarization, muscle contraction, and synaptic transmission, as they are involved in the control of neurotransmitter release from neurons [49]. Mutations in the *CACNA1A* gene are implicated as causal for spinocerebellar ataxia type 6 in humans [51]. Spinocerebellar ataxia type 6 in humans is an autosomal dominant disorder that is progressive and degenerative in nature, with an average age of onset of 43 to 52 years [51,62]. Additionally, a mouse knockout study [49] reported that *CACNA1A* knockout results in ataxia and death through the removal of channel-mediated neurotransmission. A deleterious mutation within this gene could result in altered calcium channels, which may alter presynaptic acetylcholine release, resulting in enhanced transmission in motor pathways and spasticity in the affected limb(s). Defects in many ion channels do not cause histopathological changes, thus the *CACNA1A* gene is a compelling candidate gene with respect not only to function, but also chromosomal location, as this gene is within a QTL in BTA7.

Axon and Neuron Function. *Rab3A* constitutive knockout mice (Rab3A(-/-)) are characterized by a deficiency in short and long-term synaptic plasticity. Synapses have the ability to strengthen or weaken over time in response to increases or decreases in activity, and it has been reported that *RAB3A* regulates the fusion probability of synaptic vesicles [54,55]. In mice, knocking out the *RAB3A* gene stopped activity-dependent vesicle accumulation close to and in the active zone of the presynaptic membrane when there was a depolarizing pulse in the presence of calcium [54]. These knockout mice are viable and present relatively mild phenotypes, suggesting nonessential functions of *RAB3A* [54]. Impaired vesicle recruitment was seen with repeated stimulation, and the capacity to secrete neurotransmitters, as well as vesicle docking, was impaired in *Rab3A* knockout mice [54]. A mutation in the *RAB3A* gene can result in decreased synapse strength over time due to loss of vesicle recruitment upon repeated stimulation. This could possibly lead to ataxia-related phenotypes, where ataxia is defined as an impairment of control of voluntary muscle movement [54]. Such a possibility was reported by Meilleur et al. [63], where *RAB3A* was listed as a candidate gene, though not confirmed at this time, for a familial form of spastic paraplegia. Symptoms of the familial spastic paraplegia include lower-limb spasticity and weakness. Although hind-limb weakness is generally only observed in cattle that are severely affected with spastic syndrome due to disuse atrophy of painfully affected limb(s), it is possible that mutations in the *RAB3A* gene could contribute to spasticity via a mechanism similar to that proposed for the familial spastic paraplegia reported by Meilleur et al. [63].

Additionally, *FYN* and *FIG4*, discussed previously, have functions related to either motor neuron degeneration or decreases in dendritic spine density and have been implicated in human spastic phenotypes. At this time, mutations within these genes are not thought to be causal for spastic syndrome; however, they may be indirectly implicated in spastic syndrome through defective protein–protein interactions with other proteins that can produce non-degenerative pelvic-limb spasticity.

Uncertain Functions. It is possible that the chaperone gene *bta-mir-23a-201* helps to fold proteins involved in muscle contraction. A mutated chaperone may not be able to assist with the product of a gene like *CACNA1A*, for example. In such a case, a deleterious phenotype may arise from aberrant calcium-channel folding even though the channel gene is normal. Additionally, the mutated *bta-mir-23a-201* protein could produce a mild functional calcium channel defect that is compatible with life but causes late-onset spasticity, rather than the complete removal of channel-mediated neurotransmission, which causes death in *CACNA1A* knockout mice [49]. Additionally, *ENSBTAG00000039130* could contribute to the spastic syndrome phenotype in unknown ways. Until more is known about the physiological basis of spastic syndrome, postulation into this gene function may be fruitless, as disruption in many genes can create a spastic phenotype. There is no functional evidence either for or against the involvement of this gene in spastic syndrome at this time. Based on our findings, the most promising candidate genes are located on BTA7 (*CACNA1* and *RAB3A*) and BTA9 (*FIG4*). The two genes of interest located on BTA7 are within a QTL at approximately 1–20 Mb. Additionally, the *FIG4* gene is a candidate gene and is located at approximately 41 Mb on BTA9. Mutations in *CACNA1A* could disrupt neuromuscular signaling without causing degenerative changes. Mutations in *RAB3A* could act to decrease fusion probability of synaptic vesicles in axons supplying skeletal muscles [54]. The *FIG4* gene is wholly expressed in skeletal muscle and mutations are implicated with severe neurodegenerative disorders in mice [64]. Spastic syndrome is non-degenerative, so it could be that *FIG4* mutations impair synaptic transmission through mechanisms that are non-degenerative. An investigation into *FIG4* functional rescue reports that expression of this gene in neurons is sufficient to correct the development of significant muscle atrophy [64].

Furthermore, the *CACNA1A* gene and the *FIG4* gene are both implicated as causal for progressive neurodegenerative skeletal muscular diseases in humans, and spinocerebellar ataxia type 6 and Charcot-Marie-Tooth Neuropathy Type 4J, respectively. *RAB3A* is listed as a candidate gene for a familial form of spastic paraplegia [63]. Though spastic syndrome is not degenerative, these genes may play a role in the spastic phenotype through biological functions that are currently unknown. These candidate genes share functions that could implicate them in spastic syndrome. Further research regarding the candidate gene list should be conducted to verify function in relation to spastic syndrome.

### 4.5. Implications and Further Research Recommendations

To begin with, it would be helpful to identify a single name for this disorder that could be used consistently within academia and industry. At this time, terminology such as “crampy” and “stretches” are commonly used colloquial terms, while “progressive posterior paralysis” and “periodic spasticity” have been used within academia to describe spastic syndrome. The many names for spastic syndrome have been a source of confusion for producers and scientists, which is a problem that should be minimized if a single term could be identified by consensus. Additionally, establishing a universal protocol for the diagnosis of spastic syndrome would enable facilities to make informed decisions regarding affected animals, whether it is culling or management practices while the affected bull is still serviceable. In order for consumers of bull semen to make informed decisions about whether to use semen from affected animals for their own herd, information on spastic syndrome status is necessary and should be made available. Universal diagnostic criteria would also facilitate research, for example by minimizing phenotyping errors in a future GWAS. There could be risk factors for spastic syndrome that are not yet known, such as environmental or nutritional factors that could promote the development of the disease. A follow-up study on the SNPs found significant in the GWAS needs to be performed to ascertain the genes holding or surrounding the significant SNPs found in this study. Functional and in silico analyses of these genes, with an emphasis on the location at approximately 87 Mb on BTA9 and 1–20 Mb on BTA7, will enrich knowledge of gene function related to spastic syndrome.

A GWAS using imputed whole-genome sequence data (as carried out by Chen et al. [65]) may highlight possible causal variants in the 87 Mb region of BTA9 or 1–20 Mb within BTA7, or generate new genomic regions of interest associated with spastic syndrome. With more spastic syndrome phenotypic information becoming available, a follow-up study should also be performed in which highly related affected animals are clustered for the GWAS. These GWAS results would be compared with other unrelated clustered GWAS results for possible population-specific stratification that results in novel associative variants. Additionally, genomic prediction of breeding values for spastic syndrome could be calculated. A candidate gene list was created. Additional in silico, functional, and in vivo analyses are needed to validate the genes and gene functions described in this study. The findings of this research may lead to a SNP test of risk variants that can be commercially used to test young bulls for disease risk variants for spastic syndrome within the North American Holstein population or even genomic selection after a large enough reference population is developed.

## 5. Conclusions

Spastic syndrome is a heritable disease, based on the heritability estimate derived from this study population. The genome-wide association studies using both the 50 K and the imputed high-density SNP panel indicated that spastic syndrome is likely polygenic. The most notable findings were significant SNP peaks located on BTA9 at approximately 87 Mb for all analyses, and a peak within BTA7 located at 1–20 Mb. Findings within the areas of interest in BTA7 and BTA9 were validated with random forest regression. In total, 1048 genes were mapped to significant SNPs. Functional and in silico analyses focused on genes involved in skeletal muscle and nervous system function. In particular, the *GPR126* gene has functions related to the myelination of axons, the *CACNA1A* gene is expressed at neuromuscular junctions, the *RAB3A* gene associates with synaptic vesicles until depolarization of the nerve terminal occurs, and the *FIG4* gene is only expressed in skeletal muscle. The *CACNA1A* gene is the most compelling candidate gene, as many calcium ion channel disorders are non-degenerative and produce spastic phenotypes. The candidate genes identified within this study will contribute to a further understanding of the pathophysiology of spastic syndrome. Additionally, mutant variants predicted to be deleterious within candidate genes were quantified. This study has identified regions of the genome and candidate genes that may facilitate further research into the genetic nature of spastic syndrome in North American Holsteins when larger datasets become available.

## Figures and Tables

**Table 1:** Disease status and population distribution for the genome-wide association study for spastic syndrome with two screening populations of North American Holstein cattle.

Sex	Country of Origin	Affected	Control
Male ^a^	Canada	15	9
	USA	11	5
Male ^b^	Canada	62	3
	USA	48	2
Female ^b^	Canada	19	87
	USA	2	2
Total		157	108

^a^ Forty animals obtained for the initial spastic syndrome screening population; and ^b^ 225 additional animals for second spastic syndrome screening population.

**Table 2:** Number of significant SNPs ^a^ associated with spastic syndrome including data from 265 animals genotyped with 50 K SNP panels based on the generalized quasi-likelihood score method.

Chromosome	50 K SNP Panel	Chromosome	50 K SNP Panel
BTA1	2	BTA16	1
BTA2	1	BTA17	3
BTA3	3	BTA18	4
BTA4	1	BTA19	1
BTA5	5	BTA20	6
BTA6	6	BTA21	2
BTA7	10	BTA22	13
BTA8	17	BTA23	-
BTA9	3	BTA24	-
BTA10	-	BTA25	2
BTA11	11	BTA26	3
BTA12	2	BTA27	-
BTA13	-	BTA28	-
BTA14	5	BTA29	1
BTA15	7	Total	98

^a^ Significance threshold of a maximum chromosome-wise pFDR of 10%.

**Table 3:** Top 10 significant SNPs based on the *p*-value and a maximum 10% chromosome-wise pFDR threshold criterion obtained from the 50 K data genome-wide association studies, using the generalized quasi-likelihood score method.

SNP Name	Chromosome	Location (bp)	*p*-Value	pFDR Chromosome-Wise Threshold (%)
BTA-108606-no-rs	BTA7	56,290,162	1.00 × 10^−16^	1
ARS-BFGL-NGS-34420	BTA5	115,633,531	2.22 × 10^−16^	1
BTB-00260821	BTA6	65,865,204	3.33 × 10^−16^	1
BTB-01667396	BTA6	65,889,482	4.44 × 10^−16^	1
BTB-01430347	BTA15	4,126,600	2.06 × 10^−11^	1
BTB-00815539	BTA21	34,339,514	2.41 × 10^−11^	1
BTB-01454431	BTA15	4,155,885	2.75 × 10^−11^	1
Hapmap49058-BTA-121772	BTA6	94,228,599	1.09 × 10^−10^	1
BTB-01093397	BTA8	82,016,661	1.42 × 10^−10^	1
BTB-01518195	BTA26	1,344,063	1.09 × 10^−9^	1

**Table 4:** Number of significant SNPs per chromosome in the genome-wide association study with imputed high-density (777 K) genotypes using the generalised quasi-likelihood score method, alongside SNP distributions per chromosome using various post-GWAS significance thresholds.

		Number of Significant SNPs ^a^		
Chromosome	pFDR 10%	MAF ≥ 0.2	Chi-Square Test	Homozygotic Polarity Score ≥ 0.3
BTA1	8	1	1	1
BTA2	9	1	1	1
BTA3	48	1	1	1
BTA4	3	-	-	-
BTA5	19	2	2	1
BTA6	35	-	-	-
BTA7	71	40	40	14
BTA8	103	15	14	11
BTA9	30	3	2	2
BTA10	6	1	1	1
BTA11	3	-	-	-
BTA12	6	1	1	1
BTA13	1	-	-	-
BTA14	36	24	24	18
BTA15	19	-	-	-
BTA16	20	2	2	-
BTA17	4	2	2	-
BTA18	12	2	2	2
BTA19	1	-	-	-
BTA20	35	21	19	14
BTA21	15	1	1	-
BTA22	4	1	1	-
BTA23	11	-	-	-
BTA24	2	-	-	-
BTA25	-	-	-	
BTA26	2	-	-	-
BTA27	11	-	-	-
BTA28	1	-	-	-
BTA29	6	-	-	-
Total	522	118	114	67

^a^ Significance thresholds were cumulative: initial threshold of maximum FDR chromosome-wise value of 10%, second threshold of minimum minor allele frequency (MAF) of 0.2, third threshold of a chi-square test, and fourth threshold of minimum homozygotic SNP difference of 0.3.

**Table 5:** ‘Top’ 10 significant SNPs based on the *p*-value and maximum 10% chromosome-wise pFDR threshold criteria obtained from genome-wide association studies of imputed high-density (777 K) genotypes using the generalized quasi-likelihood score method.

SNP Name	Chr.	Location (bp)	*p*-Value	pFDR Chromosome-Wise Threshold (%)	Minor Allele Frequency	Homozygotic Polarity Score
BovineHD1400022775	BTA14	80,696,596	7.81 × 10^−6^	1	0.392	0.40
BovineHD0800033791	BTA8	70,398,696	8.59 × 10^−6^	1	0.403	0.30
BovineHD4100006888	BTA8	70,445,854	8.59 × 10^−6^	1	0.403	0.30
BovineHD1400022777	BTA14	80,698,839	1.19 × 10^−5^	1	0.395	0.52
BovineHD0900010787	BTA9	38,889,327	1.21 × 10^−5^	5	0.203	0.35
BovineHD1400022780	BTA14	80,703,868	1.44 × 10^−5^	5	0.489	0.45
BovineHD1000030149	BTA10	103,183,804	1.51 × 10^−5^	5	0.479	0.42
BovineHD1400022806	BTA14	80,744,738	1.58 × 10^−5^	5	0.490	0.50
BovineHD1400022805	BTA14	80,743,593	1.59 × 10^−5^	5	0.464	0.44
ARS-BFGL-BAC-26936	BTA14	80,753,881	1.79 × 10^−5^	5	0.494	0.51

**Table 6:** Information on common significant SNPs ^a^ between 50 K and high-density (777 K) genome-wide association studies in 265 North American Holsteins screening for genomic areas of interest for spastic syndrome.

SNP Name	Chr.	Location (bp)	*p*-Value	pFDR Chromosome-Wise Threshold (%)	Minor Allele Frequency	Homozygotic Polarity Score
ARS-BFGL-NGS-67684	BTA1	46,620,571	2.98 × 10^−5^	10	0.244	0.50
Hapmap59438-rs29012637	BTA7	6,669,157	0.000475	10	0.220	0.38
ARS-BFGL-NGS-16633	BTA7	15,893,312	0.000217	10	0.259	0.30
Hapmap58018-ss46526014	BTA8	70,401,417	0.000127	5	0.403	0.32
ARS-BFGL-BAC-26470	BTA14	49,517,377	0.000175	10	0.269	0.74
BTB-01223066	BTA14	50,747,170	7.46 × 10^−5^	5	0.451	0.31
ARS-BFGL-BAC-26936	BTA14	80,753,881	1.79 × 10^−5^	5	0.263	0.50
ARS-BFGL-NGS-3276	BTA20	12,017,779	0.000332	10	0.396	0.34
ARS-BFGL-NGS-119698	BTA20	16,715,651	5.15 × 10^−5^	5	0.479	0.30
Hapmap57270-ss46526311	BTA20	16,780,563	7.59 × 10^−5^	5	0.479	0.48

^a^ Significance threshold of a maximum chromosome-wise pFDR of 10%, a minimum minor allele frequency (MAF) of 0.2, third threshold of a chi-square test, and fourth threshold of a minimum homozygotic SNP difference of 0.3.

**Table 7:** Number of significant SNPs per chromosome in the genome-wide association study with imputed high-density (777 K) genotypes using a random forest regression, alongside SNP distributions per chromosome using various post-GWAS significance thresholds.

Chromosome	Total Number of Significant SNPs	SNP Frequency Range ^a^	Average Vim% Range ^b^	Region of Interest (Mb) ^c^
BTA1	34	2–6	0.00054–0.00291	100–107
BTA2	48	2–5	0.00057–0.00248	80–90
BTA3	21	2–3	0.00068–0.00202	23–29
BTA4	78	2–5	0.00060–0.00322	110–112
BTA5	41	2–6	0.00058–0.00266	90–100
BTA6	44	2–5	0.00034–0.00340	12–15
BTA7	85	2–19	0.00056–0.00341	104–112
BTA8	19	2–5	0.00034–0.00253	60–70
BTA9	50	2–9	0.00056–0.00251	40–60
BTA10	9	2–3	0.00059–0.00173	101–103
BTA11	20	2–5	0.00028–0.00236	60–80
BTA12	46	2–6	0.00060–0.00241	30–40
BTA13	22	2–4	7.40 × 10^−19^–0.00251	27–30
BTA14	57	2–6	0.00063–0.00257	2–6
BTA15	20	2–5	0.00065–0.00202	16
BTA16	32	2–10	0.00029–0.00275	69–71
BTA17	22	2–4	0.00033–0.00220	10–11
BTA18	13	2–5	0.00061–0.00223	59
BTA19	39	2–5	0.00059–0.00251	50–58
BTA20	51	2–5	0.00028–0.00269	10–13
BTA21	13	2–5	0.00047–0.00274	65–71
BTA22	20	2–3	0.00079–0.00208	22–25
BTA23	7	2	0.00065–0.00178	38–41
BTA24	21	2–4	0.00066–0.00302	11–12
BTA25	20	2–6	0.00061–0.00198	21–28
BTA26	24	2–5	0.00065–0.00211	23–29
BTA27	18	2–7	0.00063–0.00184	31–37
BTA28	21	2–6	0.00059–0.00207	26–27
BTA29	18	2–4	0.00062–0.00254	48–50
Total	913			

^a^ Frequency of significant SNPs occurring in different forests, with a minimum forest frequency of two. ^b^ Average range of the VIM% for all significant SNPs. ^c^ Chromosomal region in which the majority of significant SNPs are located.

**Table 8:** Common SNPs between the high-density GWAS via the GQLS method and at least two forests of random forest regression for genomic areas of interest for spastic syndrome.

SNP Name	Chr.	Base Pair Location	*p*-Value	pFDR Chromosome-Wise Threshold (%)	MAF	Homozygotic Polarity Score ^a^	Average VIM%	Forest Frequency
BovineHD0700004329	BTA7	15,774,319	0.00029	10	0.45	0.20	0.00180	5
BovineHD0700003962	BTA7	14,674,895	0.00018	10	0.26	0.25	0.00087	2
BovineHD0700004239	BTA7	15,417,524	0.00054	10	0.49	0.28	0.00077	2
BovineHD0700003455	BTA7	13,282,230	0.00012	10	0.42	0.35	0.00147	4
BovineHD4100006888	BTA8	70,445,854	8.59 × 10^−6^	1	0.40	0.30	0.00201	2
BovineHD0800020415	BTA8	68,017,405	0.00062	10	0.48	0.30	0.00253	2
BovineHD0800020414	BTA8	68,016,458	0.00062	10	0.48	0.32	0.00135	2
BovineHD0800033791	BTA8	70,398,696	8.59 × 10^−6^	1	0.40	0.34	0.00098	5
BovineHD0800020416	BTA8	68,018,832	0.00062	10	0.48	0.38	0.00134	3
BovineHD1400022775	BTA14	80,696,596	7.81 × 10^−6^	1	0.39	0.40	0.00126	2
ARS-BFGL-BAC-23853	BTA14	45,174,382	0.00030	10	0.49	N/A	0.00067	2
BovineHD1400022812	BTA14	80,764,418	6.95 × 10^−5^	5	0.47	0.45	0.00160	3
BovineHD1400022779	BTA14	80,702,943	2.43 × 10^−5^	5	0.42	0.47	0.00162	2
BovineHD2000005062	BTA20	16,686,278	8.34 × 10^−5^	5	0.42	0.28	0.00119	5
BovineHD2000005856	BTA20	19,580,504	0.00010	5	0.48	0.28	0.00151	3
BovineHD2000003858	BTA20	12,044,497	0.00012	5	0.44	0.35	0.00130	2

^a^ All SNP polarity scores are significant by chi-square test.

**Table 9:** Candidate gene list for animals genotyped with imputed high-density (777 K) SNP panels, phenotyped for spastic syndrome.

Gene ID (Location, bp)	SNP ID	Chr.	SNP Location (bp)	SNP Location from Gene (bp)	Homozygous Differences ^1^	MAF	Protein Significance to Spastic Syndrome
ENSBTAG00000014828 ***CACNA1A*** (13,249,309–13,506,224)	BovineHD0700003455 BovineHD0700004239 ARS-BFGL-NGS-102773 BovineHD0700003962 BovineHD0700003008 BovineHD0700004113 BovineHD0700004235	BTA7	13,282,230 15,417,524 12,833,745 14,674,895 11,340,236 15,046,423 15,408,372	SNP is within gene 1,911,300 bases left 415,564 bases right 1,168,671 bases left 1,909,073 bases right 1,540,199 bases left 1,902,148 bases left	0.32 0.34 0.32 0.30 0.37 0.50 0.54	0.42 0.49 0.40 0.26 0.41 0.37 0.23	Adult locomotory behavior; calcium transporter for nerve impulse involved in muscle contraction
ENSBTAG00000010635 ***RAB3A*** (4,944,325–4,950,010)	BovineHD0700001946 Hapmap59438-rs29012637	BTA7	6,632,343 6,669,157	1,682,333 bases left 1,719,147 bases left	0.42 0.33	0.47 0.46	Neuron development; synaptic vesicle exocytosis
ENSBTAG00000020709 ***MAP1S*** (5,335,661–5,367,540)	BovineHD0700001946 Hapmap59438-rs29012637	BTA7	6,632,343 6,669,157	1,264,803 bases left 1,301,617 bases left	0.42 0.33	0.47 0.46	Neuron projection; development and morphogenesis
ENSBTAG00000029901 ***Bta-mir-23a-201*** (12,981,970–12,982,042)	ARS-BFGL-NGS-102773	BTA7	12,833,745	148,225 bases right	0.32	0.40	Post-translational inhibition; chaperone-like behavior
ENSBTAG00000039130 **Uncharacterized** (14,670,708–14,672,994)	BovineHD0700003737	BTA7	14,129,719	540,989 bases right	0.31	0.21	TCP-1 chaperone family; heat shock protein
ENSBTAG00000005827 ***FIG4*** (40,873,047–41,046,003)	BovineHD0900010787	BTA9	38,889,327	1,983,720 bases right	0.57	0.20	Locomotory behavior expressed in frontal lobe; where muscle movement is partially controlled
ENSBTAG00000011851 ***FYN*** (39,107,807–39,259,883)	BovineHD0900010787	BTA9	38,889,327	218,480 bases right	0.57	0.20	Locomotion; positive regulation of neuron projection development

^1^ Population proportionate distributions of homozygotic SNPs were calculated in the affected and unaffected populations, and the differences of the homozygous states were taken per disease status. Then, the absolute difference of the differences in homozygotic status was retrieved to identify the potential polarity of homozygous SNP genotypes and disease status.

**Table 10:** Consequences of SNP variants in genes of interest associated with spastic syndrome.

Gene	Missense	Synonymous	Intron Variant	Upstream Gene Variant	Downstream Gene Variant	Elongation	Truncation	Splice Region Variant
Chromosome 7								
*RAB3A*	1	2	18	25 ^a^	21	2	-	-
*MAP1S*	8 ^b^	3	119	12	30	3	4	-
*CACNA1A*	14 ^c^	21	2200	20	43	72	90	6
Uncharacterized	2 ^d^	-	16	97	78	-	2	1
Chromosome 9								
*FYN*	-	3	1115	20	17	46	54	1
*FIG4*	2	14	1496	92	80	63	77	1

^a^ One variant predicted to be deleterious. ^b^ Four variants predicted to be deleterious. ^c^ Three variants predicted to be deleterious. ^d^ One variant predicted to be deleterious.

## Data Availability

All the data needed for the interpretation of the results are provided in the paper and Supplementary Materials. The raw datasets cannot be made publicly available as they are the property of Canadian dairy farmers and commercially sensitive.

## Supplementary Materials

The following supporting information can be downloaded at: https://www.mdpi.com/article/10.3390/genes14071479/s1, Table S1: Common SNP frequencies chromosome between 50K and high density (777 K) genome-wide association studies in 265 North American Holsteins screening for genomic areas of interest for Spastic Syndrome; Table S2: Number of genes per chromosome mapped from significant SNPs from the genome-wide association study with imputed high density (777 K) genotypes, using the Next-Generation Sequencing SNP Tool; Table S3: Direct linkage of significant SNPs from GWAS and SNPS within haplotype blocks in genes of interest on chromosome 7; Table S4: Direct linkage of significant SNPs from GWAS and SNPS within haplotype blocks in the *CACNA1A* gene; Table S5: Direct linkage of significant SNPs from GWAS and SNPS within haplotype blocks in Uncharacterized gene; Table S6: Direct linkage of significant SNPs from GWAS and SNPS within haplotype blocks in the *FYN* gene; Table S7: Direct linkage of significant SNPs from GWAS and SNPS within haplotype blocks in the *FIG4* gene; Figure S1: Genome-wide Manhattan plot of the genome-wide association study with 50 K genotypes using a generalised quasi-likelihood score via Sleuth software, with significance thresholds of 1%, 5%, and 10% chromosome-wise positive false discovery rate (pFDR); Figure S2: Genome-wide Manhattan plot of the genome-wide association study with high density (777 K) genotypes using a generalised quasi-likelihood score via Sleuth software, with significance thresholds of 1%, 5%, and 10% chromosome-wise positive false discovery rate (pFDR); Figure S3: Linkage blocks (r^2^) for SNPs within the *CACNA1A* gene located on chromosome 7, obtained from Haploview. Figure S4: Linkage blocks (r^2^) for SNPs within the *RAB3A* gene located on chromosome 7, obtained from Haploview; Figure S5: Linkage blocks (r^2^) for SNPs within the *MAP1S* gene located on chromosome 7, obtained from Haploview; Figure S6: Linkage blocks (r^2^) for SNPs within the *bta-mir-23a-201* gene located on chromosome 7, obtained from Haploview; Figure S7: Linkage blocks (r^2^) for SNPs within the Uncharacterized gene located on chromosome 7, obtained from Haploview; Figure S8: Linkage blocks (r^2^) for SNPs within the *FIG4* gene located on chromosome 9, obtained from Haploview; Figure S9: Linkage blocks (r^2^) for SNPs within the *FYN* gene located on chromosome 9, obtained from Haploview.
