# Supplementary material for: Investigating the Genetic Background of Spastic Syndrome in North American Holstein Cattle Based on Heritability, Genome-Wide Association, and Functional Genomic Analyses
Source: Genes (Basel). 2023 Jul 20;14(7):1479. doi: 10.3390/genes14071479 (PMC10378964; doi:10.3390/genes14071479)
Supplement: Supplementary file 1 [file genes-14-01479-s001.zip › genes-2510439-supplementary.pdf]

SUPPLEMENTARY MATERIAL

Table S1. Common SNP frequencies chromosome between 50K and high density (777K) genome-wide association studies in 265 North American Holsteins screening for genomic areas of interest for Spastic Syndrome.

Chromosome	SNP Frequency[a]
BTA1	2 (1)
BTA2	1
BTA3	3
BTA4	1
BTA5	4
BTA6	6
BTA7	7 (2)
BTA8	17 (1)
BTA9	2
BTA10	-
BTA11	-
BTA12	2
BTA13	-
BTA14	5 (3)
BTA15	5
BTA16	1
BTA17	1
BTA18	3
BTA19	-
BTA20	5 (3)
BTA21	2
BTA22	1
BTA23	-
BTA24	-
BTA25	-
BTA26	1
BTA27	-
BTA28	-
BTA29	1
**Total**	**73 (10)**

[a] Significance threshold of a maximum 10% using chromosome-wise positive false discovery rate (pFDR). The values in brackets indicate the SNPs that remain common after all post-GWAS threshold criteria.

Table S2. Number of genes per chromosome mapped from significant SNPs from the genome-wide association study with imputed high density (777K) genotypes, using the Next-Generation Sequencing SNP Tool.

Number of Genes	
**Chromosome**	**Cohort 2 GWAS**
BTA1	28
BTA2	11
BTA3	79
BTA4	-
BTA5	146
BTA6	-
BTA7	572
BTA8	78
BTA9	44
BTA10	30
BTA11	-
BTA12	26
BTA13	-
BTA14	32
BTA15	-
BTA16	-
BTA17	-
BTA18	-
BTA19	-
BTA20	-
BTA21	-
BTA22	-
BTA23	-
BTA24	-
BTA25	-
BTA26	-
BTA27	-
BTA28	-
BTA29	-
**Total***	**1,048**

* 26 common genes between sample sizes, all located on BTA9.

Table S3. Direct linkage of significant SNPs from GWAS and SNPS within haplotype blocks in genes of interest on chromosome 7.

Gene	Chr.	Significant SNP from GWAS	SNP of interest within the haplotype block	Reference significant SNP(s)[1]	r^2	Distance between SNP pair (bp)
RAB3A	**7**	BovineHD0700001946	BTB-00292673	BovineHD0700001946	0.23	1,678,542
			BovineHD0700001415	BovineHD0700001946	0.23	1,552,950
			BovineHD0700001455	BovineHD0700001946	0.25	1,413,201
MAP1S	**7**	BovineHD0700001946<br>Hapmap59438-rs29012637	BovineHD0700001485	BovineHD0700001946	0.31	1,250,937
				Hapmap59438-rs29012637	0.31	1,287,751
			BovineHD0700001502	BovineHD0700001946	0.20	1,200,835
				Hapmap59438-rs29012637	0.20	1,237,649
bta-mir-23a-021	7	BovineHD0700003008<br>ARS-BFGL-NGS-102773<br>BovineHD0700003455<br>BovineHD0700003671<br>BovineHD0700003962	BovineHD0700003320	ARS-BFGL-NGS-102773	0.29	26,741
			ARS-BFGL-NGS-102773	BovineHD0700003008	0.25	1,493,509
				BovineHD0700003455	0.24	448,485
			BovineHD0700003327	ARS-BFGL-NGS-102773	0.39	6,005
			BovineHD0700003356	BovineHD0700003455	0.22	327,371
			BovineHD0700003360	ARS-BFGL-NGS-102773	0.22	136,063

[1] reference SNPs refer to significant variants obtained with the GWAS, and syntenic linkage (r^2) was derived from reference SNPs, and variants within candidate genes which are not identified as significant in the GWAS.

Table S4. Direct linkage of significant SNPs from GWAS and SNPS within haplotype blocks in *CACNA1A* gene.

Gene	Chr.	Significant SNP from GWAS	SNP of interest haplotype block	Reference significant SNP(s)[1]	r^2	Distance between SNP pair (bp)
CACNA1A	7	BovineHD0700003008	BovineHD0700003443	BovineHD0700003455	0.84	20,403
		ARS-BFGL-NGS-102773		BovineHD0700004239	0.52	2,155,697
		BovineHD0700003455	BovineHD0700003444	BovineHD0700003671	0.44	701,847
		BovineHD0700003671		BovineHD0700004235	0.20	2,143,929
		BovineHD0700003737	BovineHD0700003445	BovineHD0700003671	0.49	699,029
		BovineHD0700003962	BovineHD0700003446	BovineHD0700003455	0.32	14,127
		BovineHD0700004113	BovineHD0700003449	BovineHD0700004239	0.36	2,144,237
		BovineHD0700004235	BovineHD0700003452	BovineHD0700003455	0.85	5,326
		BovineHD0700004239		BovineHD0700004239	0.53	2,140,620
			BovineHD0700003453	BovineHD0700003455	0.63	688,804
				BovineHD0700004239	0.39	2,140,038
			BovineHD0700003454	BovineHD0700003455	0.82	3,215
				BovineHD0700004239	0.51	2,138,509
			Hapmap43057-BTA-80741	BovineHD0700003455	0.50	2,258
				BovineHD0700003671	0.27	686,318
				BovineHD0700004239	0.34	2,137,552
			BovineHD0700003455	ARS-BFGL-NGS-102773	0.24	448,485
				BovineHD0700003671	0.22	684,060
				BovineHD0700004239	0.51	2,135,294
			BovineHD0700003456	BovineHD0700003455	0.63	1,490
				BovineHD0700004239	0.39	2,133,804
			BovineHD0700003457	BovineHD0700003455	0.86	2,257
				BovineHD0700004239	0.53	2,133,037
			Hapmap31438-BTA-	BovineHD0700003455	0.51	24,779

			144746			
				BovineHD0700004239	0.29	2,110,515
			BovineHD0700003474	ARS-BFGL-NGS-102773	0.25	485,718
				BovineHD0700003455	0.64	37,233
				BovineHD0700004239	0.37	2,098,061

[1] reference SNPs refer to significant variants obtained with the GWAS, and syntenic linkage (r^2) was derived from reference SNPs, and variants within candidate genes which are not identified as significant in the GWAS.

Gene	Chr.	Significant SNP(s) from GWAS	SNP of interest haplotype block	Reference significant SNP(s)[1]	r^2	Distance between SNP pair (bp)
CACNA1A	7	BovineHD0700003008	Hapmap34784-BES2_Contig524_2530	BovineHD0700003455	0.20	84,514
		ARS-BFGL-NGS-102773	BovineHD0700003510	ARS-BFGL-NGS-102773	0.33	573,429
		BovineHD0700003455		BovineHD0700003671	0.25	559,116
		BovineHD0700003671	BovineHD0700003512	BovineHD0700003737	0.34	715,133
		BovineHD0700003737	BovineHD0700003513	BovineHD0700003671	0.21	548,223
		BovineHD0700003962	BovineHD0700003520	BovineHD0700003008	0.22	2,094,550
		BovineHD0700004113	BovineHD0700003553	BovineHD0700003671	0.23	426,822
		BovineHD0700004235	BovineHD4100005750	ARS-BFGL-NGS-102773	0.24	711,586
		BovineHD0700004239	BovineHD0700003563	ARS-BFGL-NGS-102773	0.23	746,034
			BovineHD0700003572	BovineHD0700003671	0.24	350,354
			BovineHD0700003573	BovineHD0700003671	0.24	348,419
			BovineHD0700003576	BovineHD0700003671	0.24	339,156
			BovineHD0700003578	BovineHD0700003671	0.24	336,568
			BovineHD0700003579	BovineHD0700003671	0.24	335,595
			ARS-BFGL-NGS-119066	BovineHD0700003671	0.24	334,116

	BovineHD0700003582	BovineHD0700003671	0.24	332,803
	BovineHD0700003590	ARS-BFGL-NGS-102773	0.26	820,783
		BovineHD0700003671	0.23	311,762
	BovineHD0700003592	ARS-BFGL-NGS-102773	0.23	835,899
		BovineHD0700003671	0.25	296,646
	BovineHD0700003596	ARS-BFGL-NGS-102773	0.24	851,436
		BovineHD0700003671	0.24	281,109

[1] reference SNPs refer to significant variants obtained with the GWAS, and syntenic linkage (r^2) was derived from reference SNPs, and variants within candidate genes which are not identified as significant in the GWAS.

Table S5. Direct linkage of significant SNPs from GWAS and SNPS within haplotype blocks in Uncharacterized gene.

Gene	Chr.	Significant SNP from GWAS	SNP of interest haplotype block	Reference significant SNP(s)[1]	r^2	Distance between SNP pair (bp)
**Uncharacterized**	7	ARS-BFGL-NGS-102773	BovineHD0700003599	BovineHD0700003671	0.27	259,067
		BovineHD0700003455		BovineHD0700004277	0.20	1,859,367
		BovineHD0700003671	BovineHD0700003891	BovineHD0700003455	0.27	1,230,419
		BovineHD0700003737		BovineHD0700003737	0.26	382,930
		BovineHD0700003962		BovineHD0700003962	0.32	162,246
		BovineHD0700004113		BovineHD0700004239	0.42	904,875
		BovineHD0700004235	BovineHD0700003940	ARS-BFGL-NGS-102773	0.21	1,797,614
		BovineHD0700004239	BovineHD0700003941	BovineHD0700003962	0.31	42,906
		BovineHD0700004277		BovineHD0700004239	0.24	785,535
			BovineHD0700003946	BovineHD0700003962	0.45	37,312
				BovineHD0700004235	0.26	770,789
				BovineHD0700004239	0.23	779,941
			BovineHD0700003951	BovineHD0700003962	0.22	27,815
				BovineHD0700004113	0.24	399,343
			BovineHD0700003954	BovineHD0700003962	0.53	22,884
				BovineHD0700004235	0.30	756,361
			BovineHD0700003959	BovineHD0700003962	0.30	6,602
				BovineHD0700004113	0.20	378,130
			BovineHD0700003960	BovineHD0700004113	0.20	377,530
				BovineHD0700004239	0.44	748,631
			BovineHD0700003964	BovineHD0700003455	0.21	1,401,256
				BovineHD0700004239	0.22	734,038
			BovineHD0700003965	ARS-BFGL-NGS-	0.22	1,853,264

		102773		
	BovineHD0700003968	BovineHD0700003962	0.47	18,818
	BovineHD0700003970	BovineHD0700004277	0.95	869,883
	BovineHD0700004000	BovineHD0700004239	0.26	656,472
	BovineHD0700004013	BovineHD0700004239	0.24	623,534
	BovineHD0700004029	BovineHD0700004239	0.25	550,665
	BovineHD0700004036	BovineHD0700004239	0.26	537,243
	BovineHD0700004038	BovineHD0700004239	0.22	533,242

[1] reference SNPs refer to significant variants obtained with the GWAS, and syntenic linkage (r^2) was derived from reference SNPs, and variants within candidate genes which are not identified as significant in the GWAS.

Table S6. Direct linkage of significant SNPs from GWAS and SNPS within haplotype blocks in *FYN* gene.

Gene	Chr.	Significant SNP from GWAS	SNP of interest haplotype block	Reference significant SNP(s)[1]	r^2	Distance between SNP pair (bp)
FYN	9	BovineHD0900010787	BovineHD0900010807	BovineHD0900010787	0.26	75,413
			ARS-BFGL-NGS-17236	BovineHD0900010787	0.23	75,880
			Hapmap48912-BTA-83311	BovineHD0900010787	0.23	103,520
			BovineHD0900010821	BovineHD0900010787	0.40	132,998
			BovineHD0900010832	BovineHD0900010787	0.30	166,779
			BovineHD0900010835	BovineHD0900010787	0.21	169,262
			BovineHD0900010844	BovineHD0900010787	0.25	184,878
			BovineHD0900010857	BovineHD0900010787	0.24	222,959
			BovineHD0900010874	BovineHD0900010787	0.23	265,254
			BovineHD0900010878	BovineHD0900010787	0.20	273,094
			BovineHD4100007378	BovineHD0900010787	0.24	313,490
			BovineHD0900010916	BovineHD0900010787	0.22	324,703

[1] reference SNPs refer to significant variants obtained with the GWAS, and syntenic linkage (r^2) was derived from reference SNPs, and variants within candidate genes which are not identified as significant in the GWAS.

Table S7. Direct linkage of significant SNPs from GWAS and SNPS within haplotype blocks in *FIG4* gene.

Gene	Chr.	Significant SNP from GWAS	SNP of interest haplotype block	Reference significant SNP(s)[1]	r^2	Distance between SNP pair (bp)
FIG4	9	BovineHD0900010787	BovineHD0900011069	BovineHD0900010787	0.266	1171008
			Hapmap59361-rs29017977	BovineHD0900010787	0.211	1199320
			BovineHD0900011091	BovineHD0900010787	0.379	1235371
			BovineHD0900011112	BovineHD0900010787	0.205	1290372
			BovineHD0900011160	BovineHD0900010787	0.244	1364642
			BovineHD4100007382	BovineHD0900010787	0.198	1388561
			BovineHD0900011171	BovineHD0900010787	0.257	1392878
			BovineHD0900011174	BovineHD0900010787	0.139	1398282
			BovineHD0900011229	BovineHD0900010787	0.201	1615060
			BovineHD0900011335	BovineHD0900010787	0.228	1903930

[1] reference SNPs refer to significant variants obtained with the GWAS, and syntenic linkage (r^2) was derived from reference SNPs, and variants within candidate genes which are not identified as significant in the GWAS.

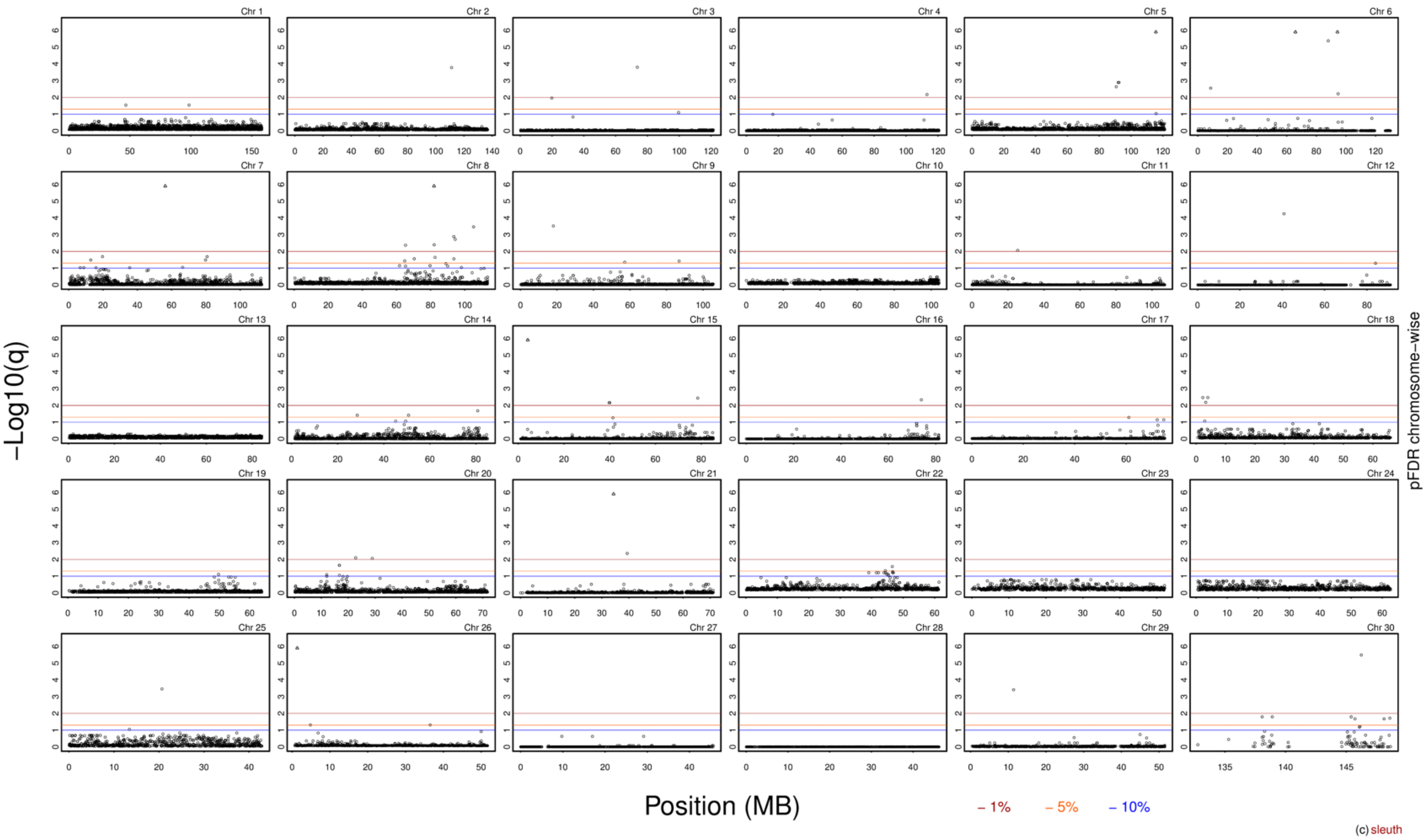

Figure S1. Genome-wide Manhattan plot of the genome-wide association study with 50K genotypes using a generalised quasi-likelihood score via Sleuth software, with significance thresholds of 1%, 5%, and 10% chromosome-wise positive false discovery rate (pFDR). Triangles indicate SNP values that exceed the –log10(q) threshold value of 5.

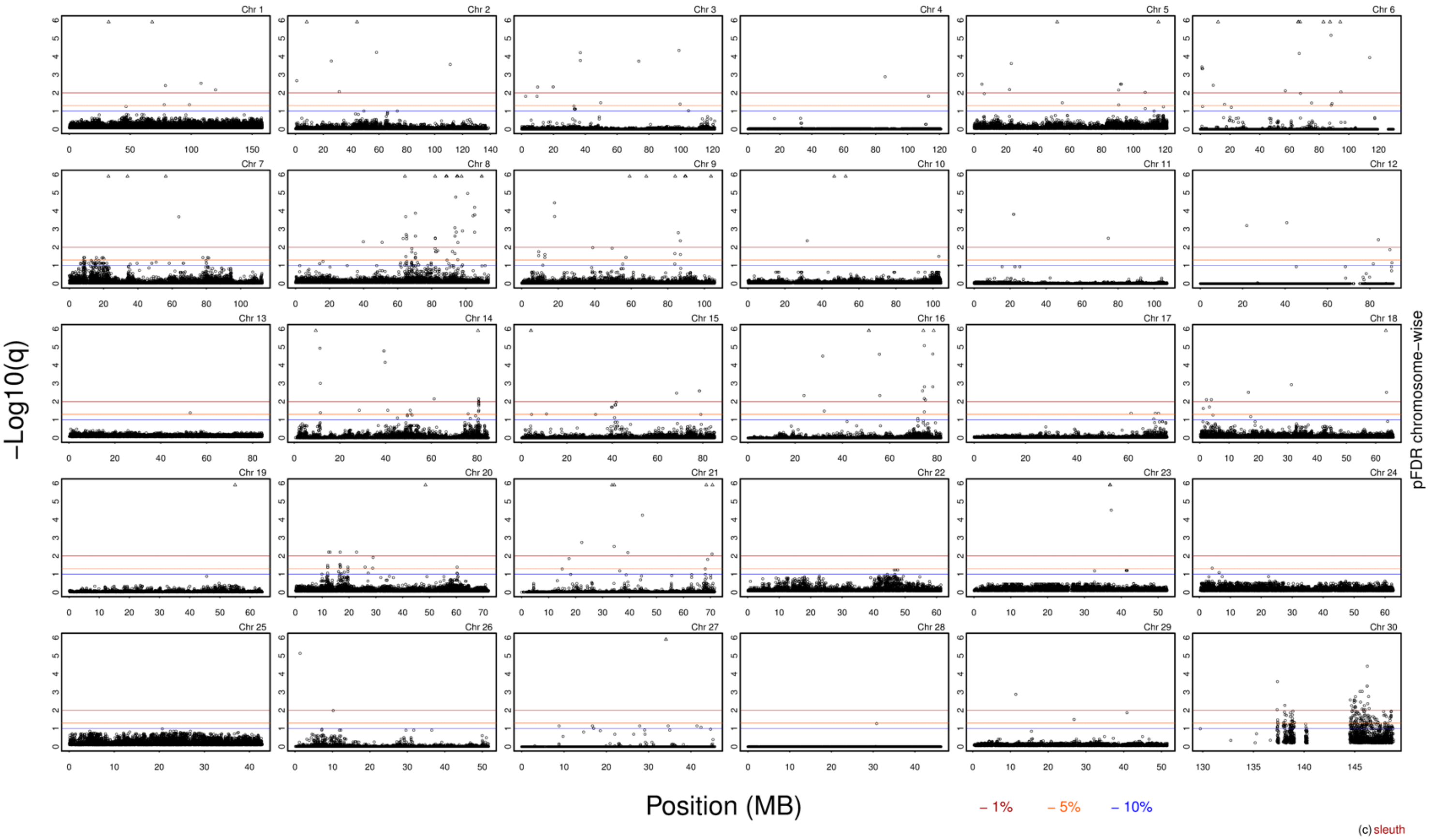

Figure S2. Genome-wide Manhattan plot of the genome-wide association study with high density (777K) genotypes using a generalised quasi-likelihood score via Sleuth software, with significance thresholds of 1%, 5%, and 10% chromosome-wise positive false discovery rate (pFDR). Triangles indicate SNP values that exceed the –log10(q) threshold value of 5.

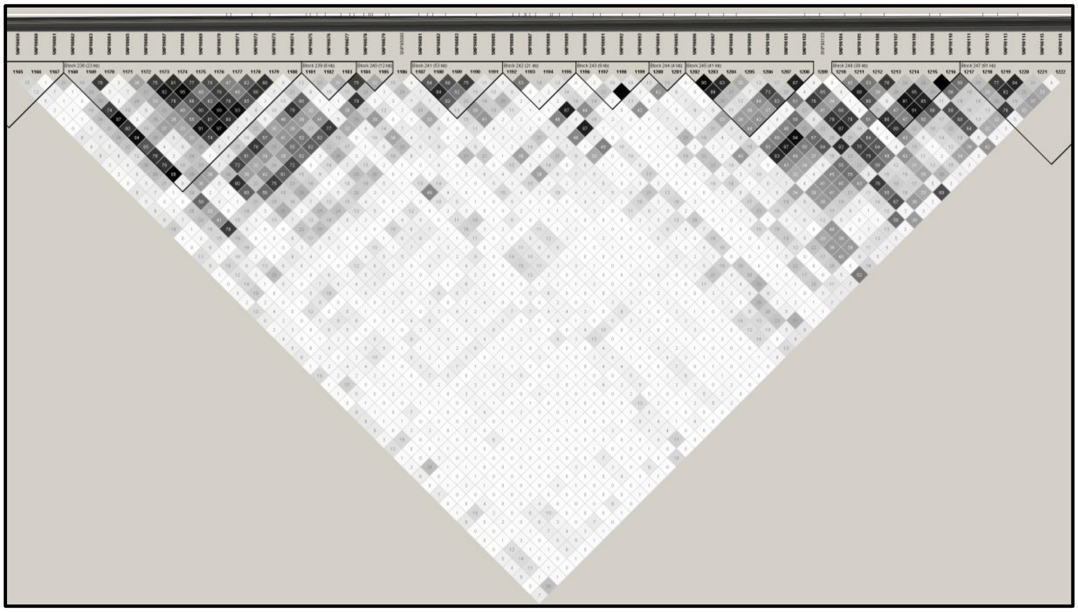

Figure S3. Linkage blocks (r^2) for SNPs within the *CACNA1A* gene located on chromosome 7, obtained from Haploview. Each diamond represents the linkage measure between two SNPs, where the darker the shade of grey, the more linkage disequilibrium (LD) is present between the two variants. Each outlined triangle represents a haplotype block measured with the solid spine method. The Solid spine method searches for a “spine” of strong LD from one marker to another, the first and the last markers within the haplotype block have strong LD to each other as well as to intermediate markers within the outlined haplotype block, although intermediate markers within the haplotype block may not be in strong LD with each other. Variant identification numbers are located at the top of the figure above the identified haplotype blocks.

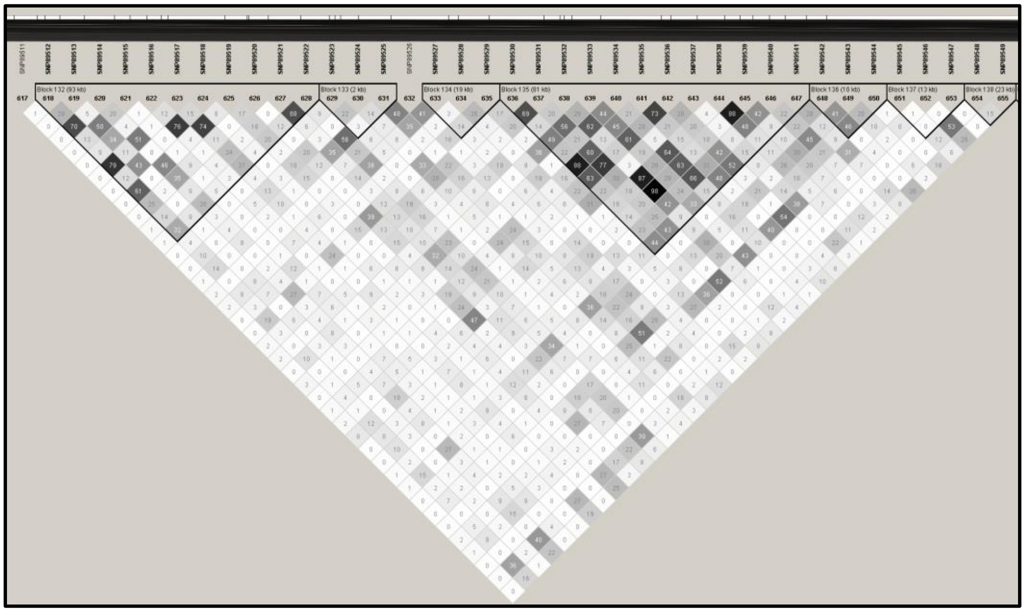

Figure S4. Linkage blocks (r^2) for SNPs within the *RAB3A* gene located on chromosome 7, obtained from Haploview. Each diamond represents the linkage measure between two SNPs, where the darker the shade of grey, the more linkage disequilibrium (LD) is present between the two variants. Each outlined triangle represents a haplotype block measured with the solid spine method. The Solid spine method searches for a "spine" of strong LD from one marker to another, the first and the last markers within the haplotype block have strong LD to each other as well as to intermediate markers within the outlined haplotype block, although intermediate markers within the haplotype block may not be in strong LD with each other. Variant identification numbers are located at the top of the figure above the identified haplotype blocks.

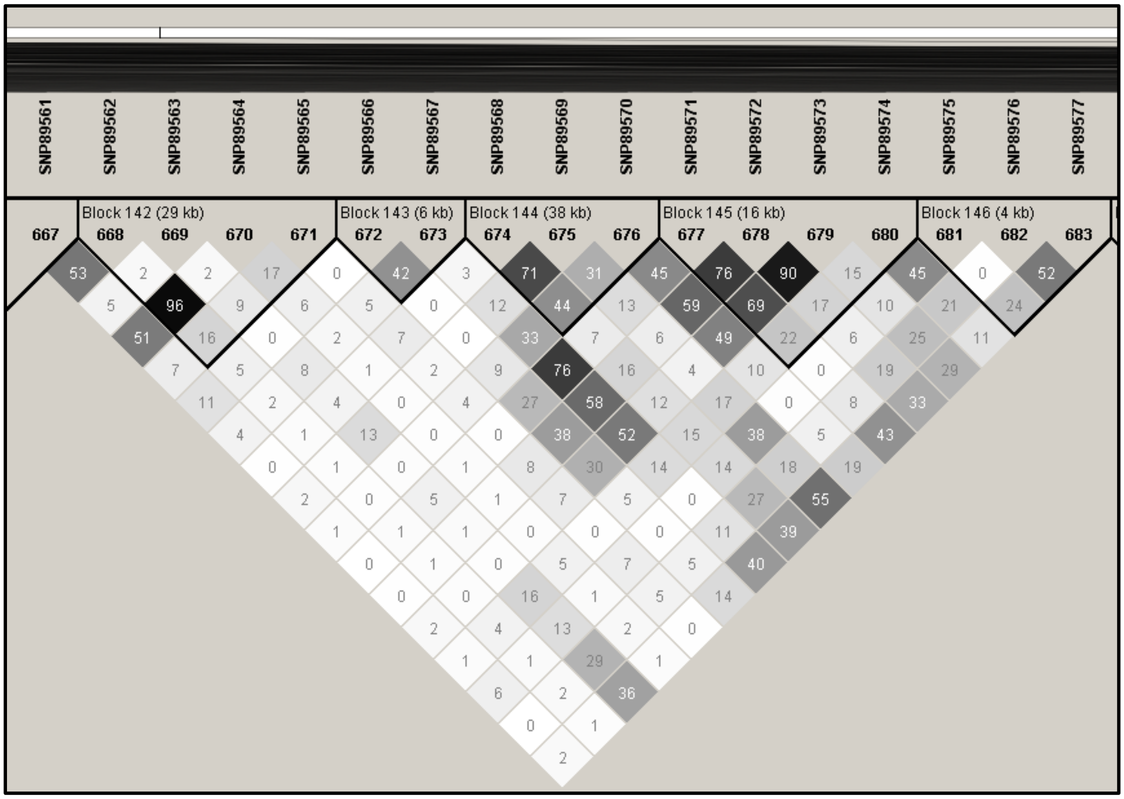

Figure S5. Linkage blocks (r^2) for SNPs within the *MAP1S* gene located on chromosome 7, obtained from Haploview. Each diamond represents the linkage measure between two SNPs, where the darker the shade of grey, the more linkage disequilibrium (LD) is present between the two variants. Each outlined triangle represents a haplotype block measured with the solid spine method. The Solid spine method searches for a "spine" of strong LD from one marker to another, the first and the last markers within the haplotype block have strong LD to each other as well as to intermediate markers within the outlined haplotype block, although intermediate markers within the haplotype block may not be in strong LD with each other. Variant identification numbers are located at the top of the figure above the identified haplotype blocks.

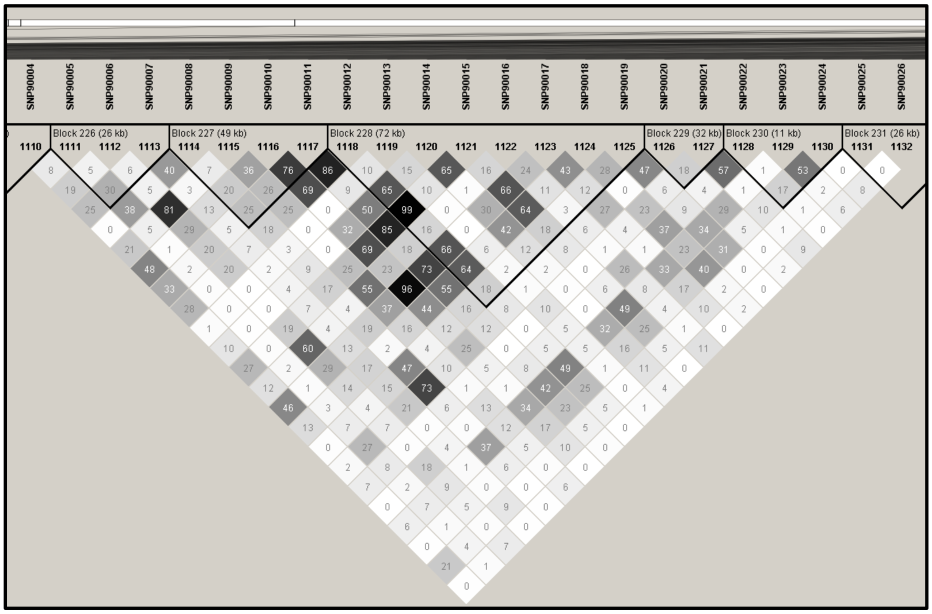

Figure S6. Linkage blocks (r^2) for SNPs within the *bta-mir-23a-201* gene located on chromosome 7, obtained from Haploview. Each diamond represents the linkage measure between two SNPs, where the darker the shade of grey, the more linkage disequilibrium (LD) is present between the two variants. Each outlined triangle represents a haplotype block measured with the solid spine method. The Solid spine method searches for a "spine" of strong LD from one marker to another, the first and the last markers within the haplotype block have strong LD to each other as well as to intermediate markers within the outlined haplotype block, although intermediate markers within the haplotype block may not be in strong LD with each other. Variant identification numbers are located at the top of the figure above the identified haplotype blocks.

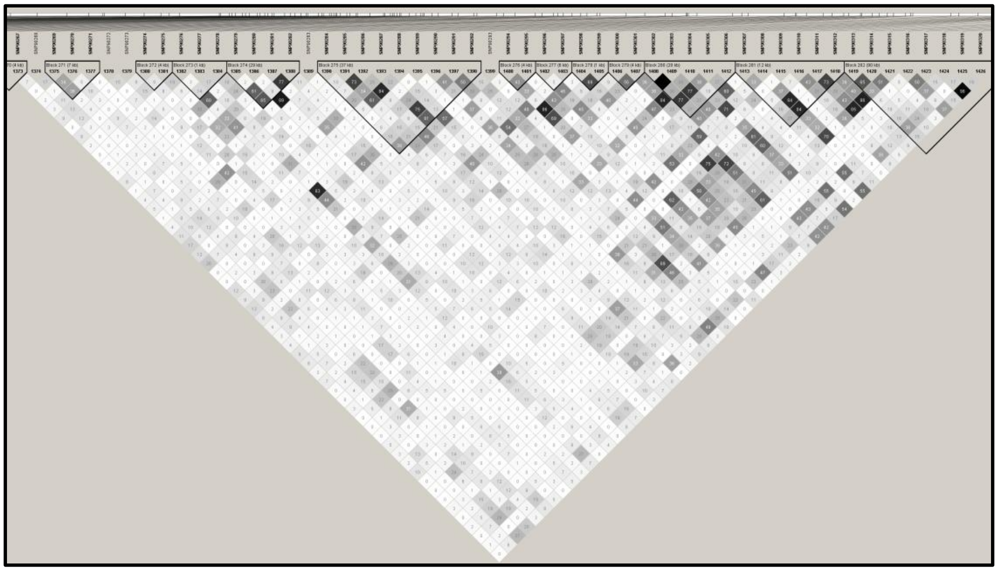

Figure S7. Linkage blocks (r^2) for SNPs within the Uncharacterized gene located on chromosome 7, obtained from Haploview. Each diamond represents the linkage measure between two SNPs, where the darker the shade of grey, the more linkage disequilibrium (LD) is present between the two variants. Each outlined triangle represents a haplotype block measured with the solid spine method. The Solid spine method searches for a "spine" of strong LD from one marker to another, the first and the last markers within the haplotype block have strong LD to each other as well as to intermediate markers within the outlined haplotype block, although intermediate markers within the haplotype block may not be in strong LD with each other. Variant identification numbers are located at the top of the figure above the identified haplotype blocks.

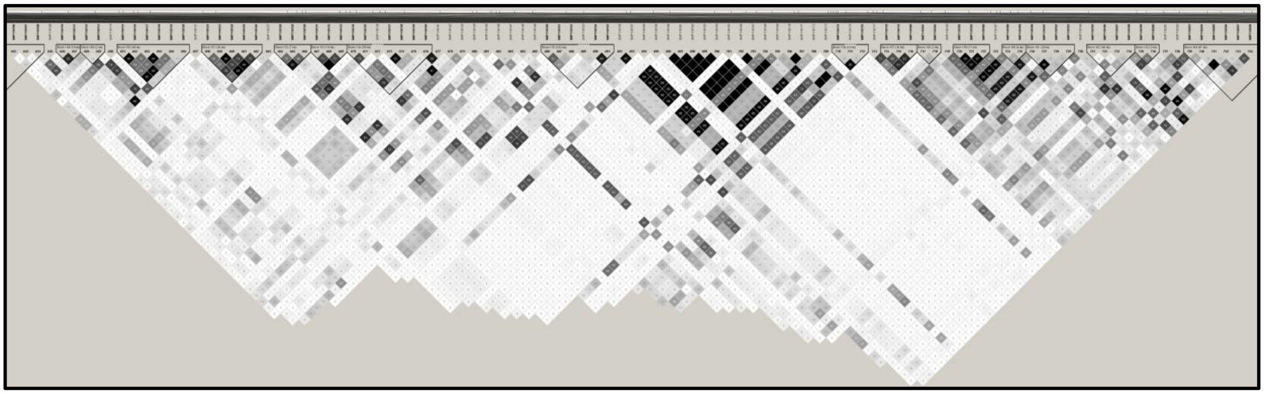

Figure S8. Linkage blocks (r^2) for SNPs within the *FIG4* gene located on chromosome 9, obtained from Haploview. Each diamond represents the linkage measure between two SNPs, where the darker the shade of grey, the more linkage disequilibrium (LD) is present between the two variants. Each outlined triangle represents a haplotype block measured with the solid spine method. The Solid spine method searches for a “spine” of strong LD from one marker to another, the first and the last markers within the haplotype block have strong LD to each other as well as to intermediate markers within the outlined haplotype block, although intermediate markers within the haplotype block may not be in strong LD with each other. Variant identification numbers are located at the top of the figure above the identified haplotype blocks.

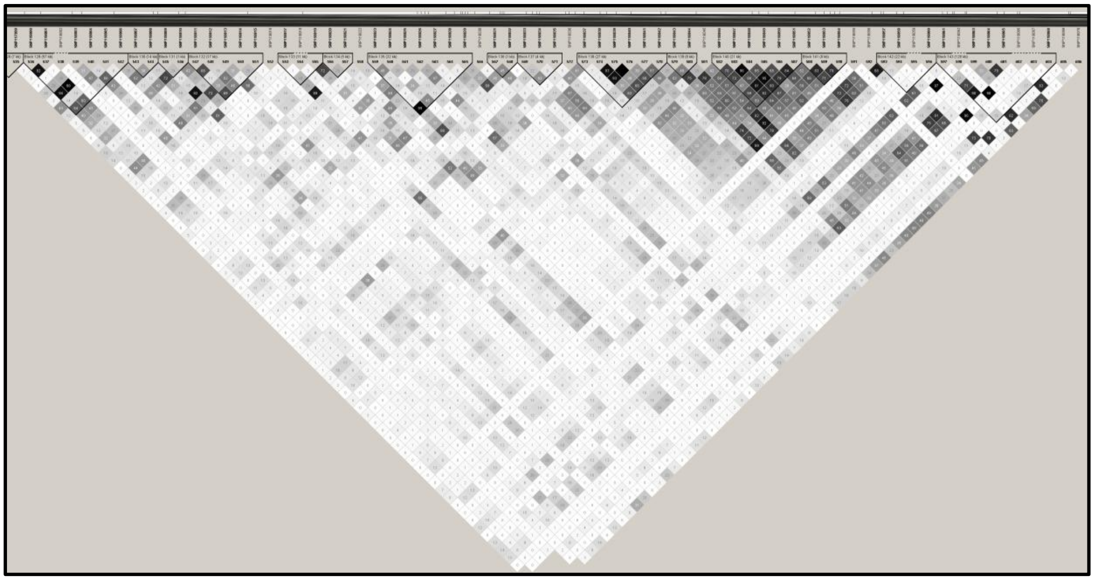

Figure S9. Linkage blocks (r^2) for SNPs within the *FYN* gene located on chromosome 9, obtained from Haploview. Each diamond represents the linkage measure between two SNPs, where the darker the shade of grey, the more linkage disequilibrium (LD) is present between the two variants. Each outlined triangle represents a haplotype block measured with the solid spine method. The Solid spine method searches for a "spine" of strong LD from one marker to another, the first and the last markers within the haplotype block have strong LD to each other as well as to intermediate markers within the outlined haplotype block, although intermediate markers within the haplotype block may not be in strong LD with each other. Variant identification numbers are located at the top of the figure above the identified haplotype blocks.
